# Quantitative proteomic analysis of sphere-forming stem-like oral cancer cells

**DOI:** 10.1186/scrt386

**Published:** 2013-12-25

**Authors:** Kaori Misuno, Xiaojun Liu, Sizhe Feng, Shen Hu

**Affiliations:** 1 School of Dentistry, University of California, Los Angeles, CA 90095, USA; 2 Jonsson Comprehensive Cancer Center, University of California, Los Angeles, CA 90095, USA

## Abstract

**Introduction:**

The purpose of this study is to identify target proteins that may play important functional roles in oral cancer stem-like cells (CSCs) using mass spectrometry-based quantitative proteomics.

**Methods:**

Sphere-formation assays were performed on highly invasive UM1 and lowly invasive UM2 oral cancer cell lines, which were derived from the same tongue squamous cell carcinoma, to enrich CSCs. Quantitative proteomic analysis of CSC-like and non-CSC UM1 cells was carried out using tandem mass tagging and two-dimensional liquid chromatography with Orbitrap mass spectrometry.

**Results:**

CSC-like cancer cells were found to be present in the highly invasive UM1 cell line but absent in the lowly invasive UM2 cell line. Stem cell markers SOX2, OCT4, SOX9 and CD44 were up-regulated, whereas HIF-1 alpha and PGK-1 were down-regulated in CSC-like UM1 cells versus non-CSC UM1 cells. Quantitative proteomic analysis indicated that many proteins in cell cycle, metabolism, G protein signal transduction, translational elongation, development, and RNA splicing pathways were differentially expressed between the two cell phenotypes. Both CREB-1-binding protein (CBP) and phosphorylated CREB-1 were found to be significantly over-expressed in CSC-like UM1 cells.

**Conclusions:**

CSC-like cells can be enriched from the highly invasive UM1 oral cancer cell line but not from the lowly invasive UM2 oral cancer cell line. There are significant proteomic alterations between CSC-like and non-CSC UM1 cells. In particular, CBP and phosphorylated CREB-1 were significantly up-regulated in CSC-like UM1 cells versus non-CSC UM1 cells, suggesting that the CREB pathway is activated in the CSC-like cells.

## Introduction

Oral cancer, predominantly oral squamous cell carcinoma (OSCC), is the sixth most common human cancer worldwide. In the United States there were 39,400 new cases of oral cavity and pharynx cancer and 7,900 related deaths in 2011. Worldwide, however, there are more than 480,000 new cases annually [1]. Patients diagnosed with OSCC often present with symptoms at a late stage, and there is a high recurrence rate after treatment, especially in those with neck lymph node metastasis [2]. Despite clinical and treatment advances, the overall five-year survival rates for oral cancer have remained low and relatively unchanged during the past few decades [3,4]. The high mortality rate of oral cancer highlights the importance of studying the molecular and cellular mechanisms of this devastating disease.

Cancer stem cells (CSCs) comprise a small subpopulation of cancer cells that possess the capacity to self-renew and to cause the heterogeneous lineages of cancer cells that comprise the tumor [5-7]. These cells appear to be responsible for tumor initiation and sustained growth, and their presence is believed to play an important role in tumor metastasis and resistance to chemotherapy or radiation therapy [8]. In this regard, studying CSC biology is critical for the discovery of novel therapeutic targets in human cancers. Enrichment and isolation of CSC-like cells have been made possible by using various methods including efflux of vital dyes by multi-drug transporters (for example, ABC transporters), increased enzymatic activity (for example, aldehyde dehydrogenase, ALDH), sphere-forming assays, and cell surface expression of specific stem cell markers [9-14]. Prince *et al*. identified a CD44^+^ subpopulation from primary head and neck squamous cell carcinomas (HNSCC) that expressed basal cell markers, cytokeratin 5 and 14, and the stem cell marker BMI1. They found that as few as 5,000 CD44^+^ HNSCC cells gave rise to a new tumor when transplanted into the flank of immunocompromised mice, whereas CD44^-^ cells failed to form tumors in the mice [15,16]. Clay *et al*. demonstrated that as few as 500 ALDH^high^ cancer cells could form new HNSCC tumors in immunocompromised mice, ten-fold fewer cells than the CD44^+^ subpopulation. Most of the ALDH^high^ cells were also CD44^+^. However, a small fraction of ALDH^+^ cells remained CD44^−^[12]. Oral CSC-like cells can also be isolated from OSCC using a sphere-formation assay by cultivating the cancer cells in serum-free medium with growth factors such as basic fibroblast growth factor (bFGF) and epidermal growth factor (EGF). Using this approach, Chiou *et al*. isolated oral CSC-like cells that expressed high levels of stem/progenitor cell markers, OCT-4, Nanog, CD117, Nestin, CD133, as well as ABC transporter ABCG2. The CSC-like cells also displayed induced differentiation abilities and enhanced migration and invasion potential. Positive correlations of OCT-4, Nanog, or CD133 expression with tumor stage were observed in OSCC patients, and Nanog/OCT-4/CD133 triple-positive patients had the worst survival prognosis [17]. Similarly, Zhang *et al*. identified a subpopulation of CD133^+^ CSC-like cells that possess higher clonogenicity, invasiveness and increased *in vivo* tumorigenicity when compared to CD133^-^ counterparts. Meanwhile, CD133^+^ CSCs were substantially resistant to standard chemotherapy, in which both *in vitro* and *in vivo* treatment with paclitaxel resulted in a marked enrichment for CD133^+^ CSCs. These findings suggest that CD133^+^ cells represent a small subpopulation of CSCs that may contribute to chemoresistance in oral cancer [13].

cAMP responsive element binding protein 1 (CREB-1) is a transcription factor that binds to the cAMP response element, a DNA nucleotide sequence present in many viral and cellular promoters. The protein belongs to the leucine zipper family of DNA-binding proteins. Various serine/threonine kinases, such as ribosomal protein S6 kinase, protein kinase C, protein kinase B/AKT, and mitogen- and stress-activated protein kinase (MSK-1), may activate CREB-1 in cells via phosphorylation [18]. Activated CREB-1 then recruits transcription co-activators CREB-1-binding protein (CBP)/p300 and relocates to the nucleus to activate a number of downstream targets, especially those genes involved in cell growth, survival and cell-cycle regulation. CREB-1 has been implicated in multiple human cancers as well as diverse cellular processes. The protein regulates a number of genes that play important roles in promoting oncogenesis, such as c-fos, cyclins A1 and cyclin D1 [19,20].

Quantitative proteomics using tandem mass spectrometry (MS) with stable isotope labeling is an important technology for measuring protein levels in a global fashion ([21]. Disease and control samples can be labeled with ‘heavy’ or ‘light’ isotopes and then measured by MS. The intensity ratios of the peptide peaks (or reporter ion peaks) in a given mass spectrum represent relative abundance of the proteins. TMT (tandem mass tag) and iTRAQ iIsobaric tag for relation and absolute quantitation) are isobaric chemical tags that enable concurrent identification and quantitation of proteins in different samples using tandem MS. The isobaric tags can be cleaved during collision-induced dissociation (CID) to yield an isotope series (reporter ions) representing the quantity of a single peptide of known mass from multiple samples. Since the peptide remains attached to the isobaric tags until CID is conducted, the peptide is simultaneously fragmented for sequence identification [22,23].

In this study, we have enriched and isolated oral CSC-like cells from a highly invasive UM1 oral cancer cell line and characterized the expression of stem cell markers in these cells. By using TMT labeling and liquid chromatography with tandem mass spectrometry (LC-MS/MS), we have performed a comparative proteomic analysis of CSC-like and non-CSC UM1 cells. Transcription co-activator CBP was found to be over-expressed whereas CREB-1 was significantly phosphorylated in CSC-like cells. Our findings suggest that the CREB-1 pathway is activated in the CSC-like UM1 cells and may play an important role in maintaining the stemness of oral CSC-like cells.

## Methods

### Cell culture

UM1 and UM2 cell lines were cultured in (D)MEM with 10% fetal bovine serum and 1% penicillin/streptomycin (Invitrogen, Carlsbad, CA, USA) and maintained at 37°C in a humidified atmosphere of 5% CO_2_. When the cancer cells reached confluence of approximately 90%, the culture medium was changed to serum-free medium ((D)MEM) containing 1% penicillin/streptomycin and growth factors bFGF (10 ng/ml) and EGF (10 ng/ml) (Geminni Bio, Sacramento, CA, USA). The spherogenic and non-spherogenic UM1 cancer cells were harvested using trypsinization.

### Western blotting

Following separation on a NuPAGENovex 4% to 12% Bis-Tris gel (Invitrogen) at 100 V, the proteins were transferred to a nitrocellulose membrane (Bio-Rad, Hercules, CA, USA). Afterwards, the membranes were blocked for one hour in 5% milk and then incubated with the following primary antibodies: rabbit polyclonal antibodies anti-CD44, anti-transketolase, anti-SOX-9, anti-CREB-1 and anti-phosphorylated CREB-1 (Santa Cruz Biotechnologies, Santa Cruz, CA, USA), rabbit polyclonal antibodies anti-SOX-2 and anti-hypoxia inducible factor 1 α (Gene Tex, Irvine, CA, USA), goat polyclonal antibody anti-OCT-3/4 and mouse monoclonal antibody anti-phosphoglycerate kinase 1 (Santa Cruz Biotechnologies). Blots were then washed with TBST (Tris-Buffered Saline with Tween 20) (1x), incubated with secondary antibody (Amersham, Piscataway, NJ, USA), and finally developed with the ECL plus detection kit (GE healthcare, Pittsburg, PA, USA).

### Tandem mass tagging

Quantitative proteomic analysis was performed using TMT labeling (TMT-6plex, Thermo Fisher, Waltham, MA, USA) and two-dimensional LC-MS/MS. CSC-like and non-CSC cells were lysed on ice with a POLYTRON homogenizer (Kenematika, Bohemia, NY, USA) in 8 M urea containing protease inhibitor cocktail (Calbiochem, San Diego, CA, USA). Equal amounts of proteins (100 μg) from either CSC-like or non-CSC UM1 cells were reduced with dithiothreitol, alkylated with iodoacetamide and digested with trypsin overnight. The resulting peptides were then labeled with TMT-126 (CSC-like) and TMT-127 (non-CSC) according to the manufacturer’s protocol. Afterwards, we combined the labeled samples and fractionated the combined sample with a strong-cation exchange spin column (VIVAPURE S mini H, Sartorius Stedim, Bohemia, NY, USA). The initial filtrate and eight elutions under different concentrations of sodium acetate (2.5 mM, 5 mM, 10 mM, 20 mM, 50 mM, 100 mM, 250 mM and 1 M) were collected, vacuum-dried and re-suspended in 0.1% formic acid for LC-MS/MS analysis.

### Liquid chromatography with tandem mass spectroscopy

Fractionated peptide samples were loaded on an Agilent nano-trap column (Santa Clara, CA, USA) and washed for 10 minutes at 6 uL/minute. Chromatography was performed using Eksigent two-dimensional-LC nanoflow system operating at 400 nL/minute and a 90-minute gradient. Separation was performed on a Microm 100 × 0.1 mm C18AQ column (200A^◦^, 3 μm) using solvent A (0.1% formic acid) and solvent B (99.9% ACN, 0.1% formic acid) over a 90-minute gradient: 0% to 30% B (60 minutes), 35% to 80% B (10 minutes), 80% B (5 minutes), and then the column was re-equilibrated. Data-dependent LC-MS-MS was performed using an Orbitrap LTQ XL mass spectrometer (Thermo Fisher, San Jose, CA, USA) with the MS scan performed in the Orbitrap analyzer using two microscans of maximum time 50 ms and an automatic gain control of 1E5. The top five ions of intensity greater than 5,000 (excluding single charge states) were selected for MS/MS. In each cycle, a MS-MS fragmentation was generated using subsequent CID (collision energy 35) and HCD (Higher energy collision dissociation) (collision energy 42) scans performed in the LTQ Iontrap and Orbitrap cell, respectively, which were then combined in data processing to obtain quantitative and qualitative data.

### Data analysis

Database searching was performed using the Proteome Discoverer 1.2 (Thermo Fisher Scientific) against the International Proteome Index (IPI) database (IPI.HUMAN.v3.16, 62322 entries). A workflow was created for the purpose of analysis of LC-MS/MS raw data. First, the raw data files were loaded into the Spectrum Files. The parameters in the Spectrum Selector were set up as default, and TMT 6-plex (TMT-126 and TMT-127) was chosen in Reporter Ion Quantifier. SEQUEST algorithm was used for data searching to identify proteins. The parameters for SEQUEST database searching were as follows: missed cleavage of two; the dynamic modifications were oxidation (+15.995 Da) (M), deamidation (+0.984 Da) (N) and phosphorylation (+79.9966 Da) (S, T, Y). The static modifications were TMT-6plex (+229.163 Da) (any N-terminus and K) and Carbamidomethyl (+57 Da) (C). The false discovery rate was below 1% for protein identification. For protein quantitation, ‘Normalize on Protein Median’ was used for normalization and proteins with a fold change of 1.2 were generally considered as differentially expressed. The gene ontology (GO) and pathway analyses were performed using the DAVID bioinformatics resource [24].

## Results

### Enrichment and isolation of CSC-like cells

**Figure 1:**
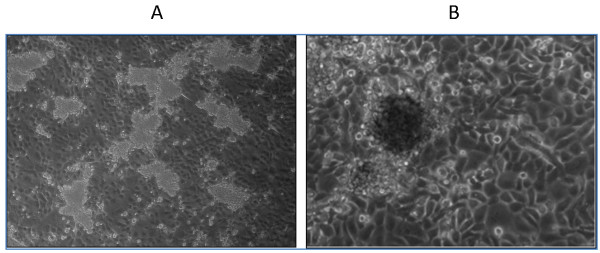
**A subset of UM1 oral cancer cells were spherogenic when cultured in serum-free medium containing growth factors bFGF and EGF for three to four weeks (A).** A close-up of the spherogenic cancer cells is shown in **(B)**. bFGF, basic fibroblast growth factor; EGF, epidermal growth factor.

To enrich and isolate CSC-like cells, both UM1 and UM2 cancer cells were maintained in serum-free media containing growth factors bFGF and EGF. After three weeks, a subset of UM1 cells started to form spherogenic aggregates, as shown in Figure 1. Significant formation of spheres was observed in the UM1 cells during the fourth week. So far, we have obtained spherogenic CSC-like cells from all 18 plates (10 cm) of UM1 cells cultured under serum-free medium (100% successful rate). In contrast, UM2 cells were not spherogenic at all in serum-free medium and lysed rapidly after three weeks. To isolate the spherogenic UM1 cells, the cultures were carefully treated with trypsin in the fifth week. The sphere-like cellular aggregates became detached from the adherent layer of cancer cells and were harvested. Afterwards, the adherent cells were collected with continuous trypsinization. The cells present in the sphere-like aggregates are considered as CSC-like cancer cells whereas the bottom layer of adherent cells are considered as non-CSC cancer cells. Both types of cells were then lysed in 8 M urea with a homogenizer for subsequent analysis of stem cell markers and quantitative proteomics. CSC-like UM1 cells were spherogenic cellular aggregates from all the twenty-one culture dishes.

### Expression of stem cell marker proteins in CSC-like cells

**Figure 2:**
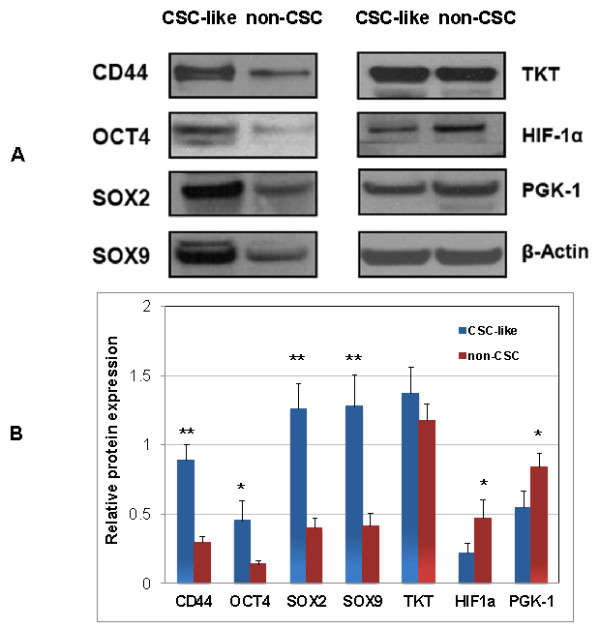
**Western blot analysis of CD44, octamer-binding transcription factor 4 (OCT4), sex determining region Y-box 2 (SOX2), SOX9, transketolase (TKT), hypoxia-inducible factor 1 alpha (HIF-α) and phosphoglycerate kinase 1 (PGK-1) in CSC-like and non-CSC UM1 cells (A).** The normalized levels of all seven proteins are shown in **(B)**. Stem cell markers CD44, OCT4, SOX2 and SOX9 were up-regulated in CSC-like UM1 cells whereas HIF-α and PGK-1 were down-regulated in CSC-like cells when compared to non-CSC UM1 cells (n = 3). No significant difference in expression was observed for TKT. *, *P* <0.05; **, *P* <0.01. CSC, cancer stem cells.

To characterize the CSC-like cells (cells present in the sphere-like aggregates), we compared the expression levels of CD44, OCT-4, SOX2 and SOX9 between CSC-like (spherogenic) and non-CSC (non-spherogenic) UM1 cancer cells with Western blotting. All the stem cell markers were significantly up-regulated in CSC-like UM1 cells when compared to non-CSC UM1 cells (Figure 2).

### Expression of HIF-1α and PGK-1 in CSC-like cells

We also compared the expression levels of hypoxia-inducible factor HIF-1α and two metabolic enzymes, transketolase (TKT) and phosphoglycerate kinase 1 (PGK-1), between CSC-like and non-CSC UM1 cells (Figure 2) with Western blotting. TKT was not significantly different between the two cell phenotypes. However, both HIF-1α and PGK1 were down-regulated in CSC-like cells versus non-CSC cells.

### Quantitative proteomic analysis of CSC-like UM1 cancer cells

**Figure 3:**
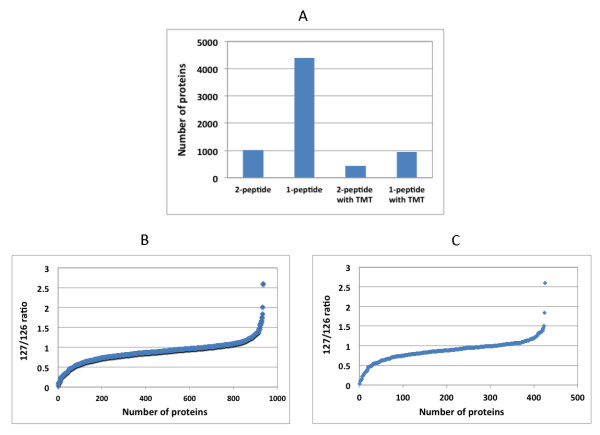
**Quantitative proteomic analysis of CSC-like and non-CSC UM1 cancer cells using tandem mass tagging (TMT) and two-dimensional LC-MS/MS.** Ratios of 127 to 126 represent the relative levels of proteins between non-CSC (TMT-127) and CSC-like (TMT-126) UM1 cells. **(A)** depicts the corresponding number of protein IDs with ≥1 matched peptide (n = 4,397), ≥2 matched peptides (n = 1,026), ≥1 matched and TMT-labeled peptide (n = 936), or ≥2 matched and TMT-labeled peptides (n = 425). **(B)** illustrates the TMT-127/TMT-126 ratios for 936 proteins with ≥1 matched and TMT-labeled peptide. **(C)** illustrates the TMT-127/TMT-126 ratios for 425 proteins with ≥2 matched and TMT-labeled peptides. CSC, cancer stem cells; LC-MS/MS, liquid chromatography-tandem mass spectroscopy.

**Figure 4:**
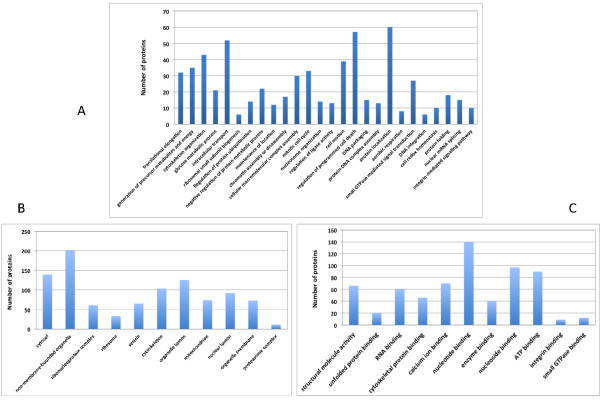
Gene ontology analysis of the quantified proteins according to the biological process (A), cellular component (B) and molecular function (C).

Using TMT labeling and two-dimensional LC-MS/MS, we performed a comparative proteomic analysis of CSC-like and non-CSC UM1 cells. The database search with the Proteome Discoverer indicated that 4,397 proteins had at least one tryptic peptide matched with high confidence and 936 peptides (21%) were labeled with TMT (Additional file 1: Table S1). There were 1,026 proteins with two tryptic peptides matched with high confidence, including 425 with two TMT-labeled peptides (Figure 3). Many proteins on cell cycle, metabolism, small G protein signal transduction, translational elongation, development, and RNA splicing pathways were found to be differentially expressed (>1.2 fold) between the CSC-like and non-CSC UM1 cells (Figure 4, Tables 1, 2, 3 and 4).

**Table 1:** A list of differentially expressed proteins regulating transcription or translation

**Accession**	**# Peptides**	**MW [kDa]**	**Calc. pI**	**127/126 non-CSC:CSC**	**SD**_**127/126**_	**Protein description**
IPI:IPI00410017.1	2	61.1	9.07	2.592	1.429	Poly(A)-binding protein 1
IPI:IPI00375141.1	5	412.1	5.83	1.456	0.421	Dystrophin
IPI:IPI00009771.5	2	69.9	5.59	1.440	0.451	Lamin-B2
IPI:IPI00291783.3	3	168.5	6.60	1.337	0.164	Gem-associated protein 5
IPI:IPI00183085.2	2	35.2	6.34	1.282	0.112	Highly similar to N-myc downstream regulated 1
IPI:IPI00179700.3	3	11.7	10.32	1.227	0.179	High mobility group protein HMG-I/HMG-Y
IPI:IPI00651769.1	2	126.6	5.63	0.832	0.140	Myelin transcription factor 1-like protein
IPI:IPI00465070.6	8	15.3	11.12	0.812	0.169	Histone H3.1
IPI:IPI00141938.3	5	12.1	10.46	0.801	0.070	H2A histone family, member V isoform 2
IPI:IPI00328737.1	2	98.6	8.40	0.794	0.110	Zinc finger protein 598
IPI:IPI00006196.2	3	236.4	5.80	0.792	0.073	Nuclear mitotic apparatus protein 1, asymmetric division related
IPI:IPI00027252.6	4	33.3	9.83	0.789	0.109	Prohibitin-2, mediator of transcriptional repression
IPI:IPI00745230.1	2	343.9	8.47	0.788	0.008	REV3-like, catalytic subunit of DNA polymerase zeta
IPI:IPI00000897.1	2	218.8	7.37	0.779	0.148	Probable helicase with zinc-finger domain
IPI:IPI00220834.7	3	82.5	5.81	0.773	0.195	ATP-dependent DNA helicase 2 subunit 2
IPI:IPI00386211.3	3	330.3	5.08	0.760	0.301	1
IPI:IPI00027107.5	5	49.8	7.61	0.760	0.136	Tu translation elongation factor, mitochondrial
IPI:IPI00021405.3	26	74.1	7.02	0.755	0.170	Isoform A of Lamin-A/C
IPI:IPI00020153.2	2	416.1	7.55	0.749	0.122	Zinc finger protein 231
IPI:IPI00219038.8	7	15.2	11.27	0.718	0.161	Histone H3.3
IPI:IPI00166612.9	2	448.9	4.78	0.711	0.063	Cardiomyopathy associated 5 protein
IPI:IPI00337766.5	3	91.7	7.39	0.705	0.215	Zinc finger, CCHC domain containing 2
IPI:IPI00642900.2	3	241.1	7.05	0.697	0.147	Transcription repressor CCR4-NOT transcription complex subunit 1
IPI:IPI00401829.3	2	93.6	6.71	0.645	0.231	Coiled-coil domain-containing protein 175
IPI:IPI00216457.6	6	14.0	10.90	0.628	0.093	Histone H2A type 2-A
IPI:IPI00065310.2	3	75.3	5.68	0.626	0.266	Coiled-coil domain-containing protein 27
IPI:IPI00014213.1	2	101.9	8.22	0.617	0.295	Probable leucyl-tRNA synthetase, mitochondrial precursor
IPI:IPI00294575.7	2	91.8	7.02	0.577	0.628	Cell division cycle protein 27 homolog
IPI:IPI00020991.2	2	61.1	9.01	0.568	0.122	CDKN2A interacting protein
IPI:IPI00171611.5	8	15.4	11.27	0.563	0.177	H3 histone family, member M
IPI:IPI00007928.4	2	273.4	8.84	0.550	0.196	Pre-mRNA-processing-splicing factor 8
IPI:IPI00021520.1	2	37.2	9.11	0.478	0.100	Glucocorticoid receptor AF-1 coactivator-1
IPI:IPI00619932.4	2	265.2	8.53	0.382	0.047	CREB-binding protein
IPI:IPI00024163.1	2	155.6	8.48	0.303	0.367	DNA-directed RNA polymerase III largest subunit
IPI:IPI00387050.3	2	55.5	9.57	0.216	0.349	BUD13 homolog, splicing factor
IPI:IPI00015557.3	2	17.7	6.01	0.097	0.248	Coiled-coil domain-containing protein 48

CSC, cancer stem cells; MW, molecular weight.

**Table 2:** A list of quantified enzymes including metabolic enzymes between CSC-like and non-CSC UM1 cells

**Accession**	**# Peptides**	**MW [kDa]**	**Calc. pI**	**127/126 non-CSC:CSC**	**SD**_**127/126**_	**Protein description**
IPI:IPI00219568.3	5	44.6	8.54	1.389	0.190	Phosphoglycerate kinase, testis specific (PGK2)
IPI:IPI00289800.7	2	59.2	6.84	1.339	0.014	Glucose-6-phosphate dehydrogenase
IPI:IPI00005705.1	2	37.0	6.54	1.258	0.161	Serine/threonine-protein phosphatase 1, catalytic subunit, gamma isozyme
IPI:IPI00218353.3	3	121.0	7.75	1.210	0.287	Probable cation-transporting ATPase 13A1
IPI:IPI00007188.4	7	32.7	9.74	0.866	0.085	ADP/ATP translocase 2
IPI:IPI00027442.3	2	106.7	5.49	0.861	0.226	Alanyl-tRNA synthetase
IPI:IPI00440493.2	9	59.7	9.13	0.824	0.161	ATP synthase alpha chain, mitochondrial precursor
IPI:IPI00029144.1	2	130.2	5.21	0.818	0.055	Serine/threonine-protein phosphatase 2A 72/130 kDa regulatory subunit B
IPI:IPI00014375.1	2	109.2	5.50	0.790	0.164	Glutamyl aminopeptidase
IPI:IPI00513928.1	2	33.0	8.15	0.768	0.076	Acyl-coenzyme A thioesterase 2
IPI:IPI00018206.3	3	47.4	9.01	0.734	0.141	Aspartate aminotransferase, mitochondrial precursor
IPI:IPI00291006.1	8	35.5	8.68	0.732	0.194	Malate dehydrogenase, mitochondrial precursor
IPI:IPI00297084.7	2	50.8	6.55	0.731	0.011	Dolichyl-diphosphooligosaccharide-protein glycosyltransferase
IPI:IPI00303476.1	15	56.5	5.40	0.730	0.203	ATP synthase beta chain, mitochondrial precursor
IPI:IPI00022774.2	4	89.1	5.26	0.665	0.078	Transitional endoplasmic reticulum ATPase
IPI:IPI00438875.2	2	98.0	7.34	0.658	0.281	Channel kinase 2 (Isoform M6-kinase 2 of Transient receptor potential cation channel subfamily M member 6)
IPI:IPI00640240.1	2	58.6	9.13	0.648	0.149	Serine palmitoyltransferase, long chain base subunit 2-like
IPI:IPI00646947.1	3	95.1	5.77	0.626	0.222	Highly similar to Sarcoplasmic/endoplasmic reticulum calcium ATPase 1
IPI:IPI00009634.1	4	49.9	9.11	0.546	0.198	Sulfide:quinone oxidoreductase, mitochondrial precursor
IPI:IPI00657779.1	2	16.5	9.58	0.492	0.172	Peptidylprolyl isomerase F
IPI:IPI00552972.2	3	56.3	6.33	0.473	0.208	Dolichyl-diphosphooligosaccharide-protein glycosyltransferase subunit 2
IPI:IPI00658151.1	3	354.1	8.51	0.465	0.276	Striated muscle preferentially expressed protein kinase
IPI:IPI00022314.1	5	24.7	8.25	0.400	0.263	Superoxide dismutase [Mn], mitochondrial precursor
IPI:IPI00007611.1	3	23.3	9.96	0.353	0.267	ATP synthase,subunit O, mitochondrial precursor

CSC, cancer stem cells; MW, molecular weight.

**Table 3:** A list of quantified proteins related to G protein signal transduction

**Accession**	**# Peptides**	**MW [kDa]**	**Calc. pI**	**127/126 non-CSC:CSC**	**SD**_**127/126**_	**Protein description**
IPI:IPI00009342.1	9	189.1	6.48	0.938	0.206	Ras GTPase-activating-like protein IQGAP1
IPI:IPI00183572.3	3	174.0	7.02	0.884	0.415	Dedicator of cytokinesis protein 7, guaninenucleotide exchange factor
IPI:IPI00383449.2	3	23.5	9.95	0.884	0.115	Ras-related protein Rab-15
IPI:IPI00337802.3	3	100.9	7.71	0.867	0.273	Disheveled-associated activator of morphogenesis 1
IPI:IPI00015148.3	2	20.8	5.78	0.852	0.051	Ras-related protein Rap-1b
IPI:IPI00016342.1	4	23.5	6.70	0.848	0.143	Ras-related protein Rab-7
IPI:IPI00746773.1	2	179.4	7.66	0.804	0.100	IQ motif containing GTPase activating protein 3
IPI:IPI00216989.5	3	82.4	7.69	0.740	0.125	G protein-regulated inducer of neurite outgrowth 3
IPI:IPI00008964.3	4	22.2	5.73	0.733	0.164	Ras-related protein Rab-1B
IPI:IPI00016513.5	3	22.5	8.38	0.727	0.120	Ras-related protein Rab-10
IPI:IPI00607850.1	2	176.9	6.58	0.654	0.072	Citron Rho-interacting kinase
IPI:IPI00168769.6	3	222.4	7.81	0.647	0.244	Exophilin 5 (Rab GTPase binding protein)
IPI:IPI00455852.1	4	91.9	8.25	0.642	0.234	Rho guanine nucleotide exchange factor 15
IPI:IPI00022479.4	3	531.9	6.04	0.554	0.264	Guanine nucleotide exchange factor p532

CSC, cancer stem cells; MW, molecular weight.

**Table 4:** A list of differentially expressed proteins related to development or differentiation

**Accession**	**# Peptides**	**MW [kDa]**	**Calc. pI**	**127/126 non-CSC:CSC**	**SD**_**127/126**_	**Protein description**
IPI:IPI00031547.1	3	107.4	5.00	0.146	0.158	Desmoglein-3 precursor
IPI:IPI00027462.1	6	13.2	6.13	0.297	0.224	Protein S100-A9
IPI:IPI00746049.1	3	131.1	6.20	0.326	0.212	Similar to Breast cancer antigen NY-BR-1.1
IPI:IPI00007047.1	2	10.8	7.03	0.351	0.186	Protein S100-A8
IPI:IPI00619932.4	2	265.2	8.53	0.382	0.047	CREB-binding protein
IPI:IPI00027412.4	2	37.2	5.82	0.470	0.086	Carcinoembryonic antigen-related cell adhesion molecule 6
IPI:IPI00168698.1	2	128.5	6.09	0.470	0.136	PDZ domain-containing protein 8
IPI:IPI00657779.1	2	16.5	9.58	0.492	0.172	Peptidylprolyl isomerase F
IPI:IPI00646867.1	4	49.6	5.25	0.555	0.142	Vimentin
IPI:IPI00168913.1	2	139.8	6.65	0.738	0.269	Limbin, positive regulator of the hedgehog signaling pathway
IPI:IPI00179700.3	3	11.7	10.32	1.227	0.179	High mobility group protein HMG-I/HMG-Y
IPI:IPI00021812.1	2	312.3	6.73	1.234	0.320	Neuroblast differentiation-associated protein AHNAK
IPI:IPI00183085.2	2	35.2	6.34	1.282	0.112	Highly similar to N-myc downstream regulated 1
IPI:IPI00410096.1	3	197.5	6.13	1.333	0.267	Selective LIM binding factor homolog
IPI:IPI00414203.2	3	81.4	5.97	1.351	0.265	Predicted testis protein
IPI:IPI00220709.3	9	33.0	4.67	1.386	0.160	Isoform 2 of Tropomyosin beta chain
IPI:IPI00220827.4	3	4.9	5.36	1.392	0.108	Thymosin beta-10
IPI:IPI00009771.5	2	69.9	5.59	1.440	0.451	Lamin-B2
IPI:IPI00375141.1	5	412.1	5.83	1.456	0.421	Dystrophin, morphogenesis and DNA-dependent regulation of transcription
IPI:IPI00005750.1	3	255.3	9.11	1.840	0.499	Polycystic kidney disease and receptor for egg jelly-related protein

CSC, cancer stem cells; MW, molecular weight.

### CREB pathway is activated in CSC-like UM1 cancer cells

**Figure 5:**
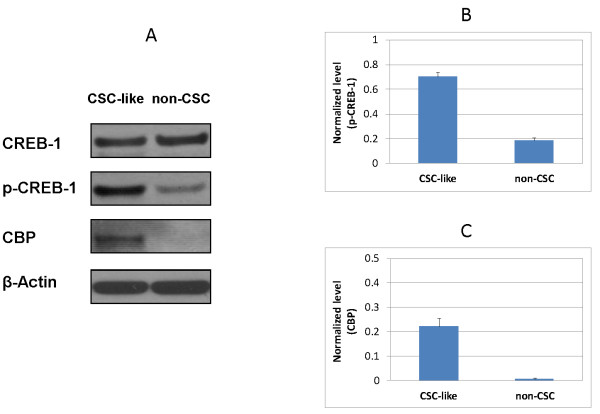
**Western blot analysis of CREB-1, phospho-CREB1 (p-CREB-1) and CREB-binding protein (CBP) between CSC-like and non-CSC UM1 cells (A).** The normalized level of p-CREB-1 or CBP against β-actin is shown in **(B)** and **(C)**. Both CBP and p-CREB-1 were significantly (*P* <0.0005, n = 3) over-expressed in CSC-like UM1 cells when compared to non-CSC UM1 cells. CSC, cancer stem cells.

Quantitative proteomic analysis revealed that CBP was over-expressed in CSC-like cells when compared to non-CSC UM1 cells. This was confirmed by Western blot analysis, as shown in Figure 5. Intriguingly, although no significant difference in the total expression level of CREB-1 was observed between non-CSC and CSC-like cells, the expression level of phosphorylated CREB-1 (p-CREB-1) was significantly higher in CSC-like UM1 cells than in non-CSC UM1 cells.

## Discussion

To enrich CSCs from oral cancer cell lines, we maintained UM1 and UM2 oral cancer cells in serum-free culture medium with bFGF and EGF. Both UM1 and UM2 cell lines were initially established from the same tongue cancer patient [25]. UM1 cells appeared to be highly invasive whereas UM2 cells were found to be lowly invasive. Our trans-well invasion assays indeed demonstrated that UM1 cancer cells were at least eight-fold more invasive than UM2 cancer cells (data not shown). After being maintained in serum-free medium for three weeks, a subset of UM1 cells started to form sphere-like cellular aggregates (CSC-like). It is well known that serum-free medium with selected growth factors is capable of maintaining cancer cells in the undifferentiated state. In fact, CSC-like cells enriched with serum-free medium containing bFGF and EGF mirror the phenotype and genotype of primary tumors more closely than do cancer cell lines cultured in complete serum medium [26]. Our experiment agreed with these previous studies and further confirmed that oral CSC-like cells can be enriched with sphere-formation assay under serum-free culture conditions [13,17]. In contrast, the UM2 tongue cancer cells were not spherogenic and eventually lysed after three weeks of culture in serum-free medium. UM1 and UM2 cell lines were derived from the same patient’s tongue squamous cell carcinoma. Our studies suggest that a CSC-like subpopulation is only present in highly invasive UM1 cancer cells but absent in the lowly invasive UM2 cancer cells.

To confirm that the spherogenic UM1 cells are CSC-like, we compared the expression levels of stem cell markers, including CD44, OCT4, SOX2 and SOX9, between the isolated spherogenic and non-spherogenic UM1 cells. All these markers were found to be significantly up-regulated in spherogenic cells (Figure 2). CD44 is a CSC marker known to be involved in cancer cell adhesion and migration. The expression of CD44 has been correlated with tumor progression and poor diagnosis in oral/head and neck cancer. Prince *et al*. purified CSC-like cells from HNSCC by selecting the CD44^+^ cells [15,16]. The CD44+ cells were found to be tumorigenic and differentially expressed the stem cell marker gene BMI1, at both the RNA and protein levels. In the study by Chiou *et al*., CD44 was found to be down-regulated whereas CD133 was over-expressed in their enriched CSC-like cells. As the authors pointed out, this may be due to the fact that the serum-free cultivation mainly enriched the CD133^+^ CSCs [17].

OCT4 and SOX2 are frequently used as markers for undifferentiated cells. OCT-4 is critically involved in the self-renewal of undifferentiated embryonic stem cells as evidenced by the fact that gene knockdown of OCT4 promotes differentiation in human embryonic stem cells [27]. Previous studies also indicated that higher OCT4 expression correlates with oral cancer progression and metastasis, as well as contributes to oral cancer patient mortality. It is speculated that deregulation of *Oct-4* gene may perturb the normal differentiation program and predispose to tumor formation [28]. SOX2 is a member of the Sox family of transcription factors, which serves as a key regulator of stem cell pluripotency and differentiation. The LIF (leukemia inhibitory factor) signaling pathway, which maintains pluripotency in mouse embryonic stem cells, activates SOX2 downstream of the JAK-STAT signaling pathway and Nanog downstream of PI3K-AKT, respectively, and maintains the expression of OCT4 [29]. As a feedback mechanism, both OCT4 and SOX2 positively regulate the transcription of pluripotency circuitry proteins in the LIF signaling pathway. In fact, SOX2 is up-regulated in lung squamous cell carcinoma, and activates cellular migration and anchorage-independent growth [30]. Similar to SOX2, SOX9 plays a pivotal role in many stages of mammalian development, with its levels strictly controlled for normal embryogenesis. The expression of SOX9 is up-regulated in a variety of human cancers, which correlates with tumor malignancy and progression. In particular, SOX9 promotes proliferation and prevents differentiation in intestinal stem cell/progenitors, acting downstream of Wnt signaling [31]. Overall, the significant over-expression of stem cell markers SOX2, OCT4, SOX9 and CD44 suggests that the cells present in spherical aggregates are CSC-like whereas the non-spherogenic, adherent layer of cells are non-CSC cancer cells.

We also compared the expression of HIF-1α and two metabolic enzymes PGK1 and TKT between CSC-like and non-CSC cells. TKT, a critical metabolic enzyme of the pentose phosphate pathway, was not significantly different between the two cell types. However, both HIF-1α and PGK1 were found to be down-regulated in CSC-like cancer cells compared to non-CSC cancer cells. HIF-1 is a heterodimeric transcription factor that consists of two subunits, the HIF-1β subunit which is constitutively expressed and the HIF-1α subunit which is regulated by oxygen levels. The protein plays an important role in cancer cell metabolism by activating the genes for glucose transporters and glycolytic enzymes resulting in an increased glycolytic rate [32]. PGK1 is an ATP-generating enzyme in the glycolytic pathway regulated by HIF-1α. Recent studies suggest that prostate cancer cell-derived PGK-1 induces osteoblastic differentiation of bone marrow stromal cells, affecting bone formation at the metastatic site. PGK-1 may also induce the differentiation of gastric cancer stem cells [33,34]. Since HIF-1α positively regulates glycolytic enzymes, including PGK1, it is not surprising that PGK-1 was down-regulated, the same as HIF-1α, in CSC-like UM1 cells.

Functional analysis of the differentially expressed proteins using DAVID revealed multiple pathways significantly associated with CSCs, such as ribosome, cell cycle, development, glycolysis, translation elongation, proteasome, small G protein signal transduction and RNA splicing. GO analysis of the quantified proteins showed similar functional categories as the pathway analysis (Figure 4). A number of the differentially expressed proteins are transcription factors/activators/suppressors, translation elongation factors, or spicing factors (Table 1). For instance, both prohibitin-2 (PHB2) and lamin A were found to be up-regulated whereas Lamin-B2 was down-regulated in CSC-like UM1 cells. PHB2 acts as a mediator of transcriptional repression by nuclear hormone receptors via recruitment of histone deacetylases. Recent studies have demonstrated that epidermal stem cells are highly dependent on PHB2 for maintaining mitochondrial function [35]. Nuclear lamins are fibrous proteins which interact with membrane-associated proteins to form the nuclear lamina on the interior of the nuclear envelope. They are involved in transcriptional regulation, breakdown and reformation of the nuclear envelope during mitosis, as well as the positioning of nuclear pores. In fact, colorectal cancer patients expressing lamin A/C in their tumor tissue were found to have a higher risk of mortality compared to patients with lamin A/C-negative tumors. The poor outcome associated with lamin A/C-positive tumors might be reflective of a more stem-cell-like phenotype [36,37].

The proteomic analysis suggests that metabolic pathways may be different between the CSC-like and non-CSC UM1 cells (Table 2). ATP synthase (α-, β-, and O-subunits) was found to be over-expressed in CSC-like cells compared to non-CSC UM1 cells. ATP synthase is an important enzyme that provides cells with energy through the synthesis of ATP. Down-regulation of ATP synthase β-subunit expression was reported in liver, kidney, colon, squamous esophageal and lung carcinomas, as well as in breast and gastric adenocarcinomas, indicating that bioenergetic dysfunction of mitochondria is a hallmark of these types of cancers [38-40]. However, in our present study, mitochondrial ATP synthase was up-regulated in CSC-like UM1 cells, suggesting an increase of mitochondrial bioenergetic production in CSC-like cells when compared to non-CSC cells. This is in agreement with the observation of a compromised glycolysis as indicated by down-regulation of HIF-1α and glycolytic enzyme PGK1 (Figure 2). Together these results seem to suggest that there is a shift from glycolytic to mitochondrial bioenergetic production in CSC-like cells when compared to non-CSC cells.

Our study also indicates that a group of small G proteins were over-expressed in CSC-like cells versus non-CSC cells (Table 3). Previous studies, indeed, have revealed important roles for G proteins and G protein-coupled receptors in regulating stem cell function. For instance, signaling mediated by G proteins has been shown to regulate pluripotency/differentiation in mouse embryonic stem cells or human hematopoietic stem cells and affect the morphology and organization of inducible pluripotent stem cell colonies [41-43]. In addition, many of the pathways downstream of G protein signaling directly regulate, or are synergistic with, the pathways that are critical in regulating stem cell pluripotency and differentiation, such as SMAD/Nanog signaling, PI3K/AKT, MAP kinase/cJun/cFos as well as GSK3/β-catenin [44]. Thus, it is not surprising that small G proteins are implicated as regulators of pluripotency and differentiation in diverse stem cell populations.

Lastly, a number of differentially expressed proteins identified by proteomic analysis may have functions related to development or differentiation (Table 4). For instance, we found that the expression of carcinoembryonic antigen-related cell adhesion molecule 6 (CEACAM6) was elevated in CSC-like UM1 cells. Previous studies indeed demonstrated that CEACAM6 is a novel marker for colorectal cancer stem cells [45] and may serve as a potential therapeutic target for pancreatic adenocarcinoma [46]. Significant over-expression (seven-fold) of desmoglein 3 (DSG3) was also observed in CSC-like UM1 cells. The functional role and clinical utility of DGS3 in head and neck cancer have been studied previously. Chen *et al*. found that the expression levels of DSG3 were correlated with clinicopathologic features of head and neck tumors and DSG3-positive cancer cells represented a more aggressive cancer phenotype [47]. Ferris *et al*. discovered that DSG3 (also known as, pemphigus vulgaris antigen) is a highly valuable biomarker for detection of lymph node metastasis in head and neck cancer. Combining sentinel node biopsy with qPCR testing of DSG3/PVA may allow intraoperative staging of HNSCC for treatment decision-making [48-50]. In addition, we found that vimentin, which is a strong indicator of epithelial-mesenchymal transition (EMT), was up-regulated in spherogenic UM1 cells versus non-spherogenic cells (non-CSC:CSC-like = 0.555, Table 4). This is in line with previous studies which demonstrated that gain of EMT promotes the stemness properties of cancer cells. The activation of EMT programs has been associated with the acquisition of stem cell traits by normal and neoplastic cells. The connection between EMT and stem cells seems to indicate that epithelial stem cells express a wide array of mesenchymal markers and EMT programs would seem to provide a ready source of CSCs by enabling the dedifferentiation of the more epithelial cells within carcinomas [51-53].

Our study revealed that the CREB pathway is activated in CSC-like UM1 cells. CREB-1 can be activated by various cellular kinases including ribosomal protein S6 kinase pp90RSK [54], protein kinase C [55], AKT/protein kinase B [56], mitogen- and stress-activated protein kinase (MSK-1) [57], MAPKAP-2 [58], and calcium/calmodulin kinases [59], subsequently activating CREB in cells. The crucial event in the activation of CREB is the phosphorylation of Ser133 in KID (kinase-inducible domain), which triggers the recruitment of transcriptional co-activators such as CBP and p300 [18]. In the present study, we found that CBP was over-expressed and CREB-1 was highly phosphorylated in spherogenic UM1 cells, suggesting that the CREB pathway is activated in these CSC-like cells. Since the cells were cultured in serum-free medium containing bFGF and EGF, very likely these growth factors up-regulated the expression of kinases such as MSK-1 [18] in a small subset of UM1 cancer cells (CSC-like cancer cells) and subsequently activated CREB-1. However, why EGF and bFGF activate the CREB pathway only in a specific subset of CSC-like UM1 cells but not the other non-CSC cancer cells remains to be further elucidated.

## Conclusions

As a summary, we have found that the highly invasive UM1 oral cancer cell line harbors a subset of CSC-like cancer cells whereas the low invasive UM2 oral cancer cell line does not. By using serum-free medium containing the growth factors bFGF and EGF for cell culture, we were able to enrich and harvest CSC-like UM1 cancer cells. Stem cell markers such as SOX2, OCT4, SOX9 and CD44 were found to be up-regulated whereas HIF-1α and PGK1 were down-regulated in the CSC-like cells compared to non-CSC cells. Comparative proteomic analysis revealed that many proteins on cell cycle, metabolism, small G protein signal transduction, translational elongation, development, and RNA splicing pathways are differentially expressed between the two cell phenotypes. In particular, we found CBP was over-expressed and CREB-1 was heavily phosphorylated in CSC-like cells. This suggests that the CREB pathway is activated and may play an important role in maintaining the stemness in the CSC-like cancer cells. There have been few studies on the role of the CREB pathway in stem cell function. Cheng *et al*. found that CREB is highly expressed in lineage negative hematopoietic stem cells and serves as a critical regulator of normal hematopoiesis and leukemogenesis [60]. To the best of our knowledge, this is the first study reporting a potential role of CREB-1 in solid tumor stem-like cells. However, the main weakness of the current study is that we have not demonstrated that the enriched CSC-like UM1 cells are capable of initiating *in vivo* tumors. In the future, we will use a xenografted transplantation assay to study if spherogenic UM1 cells enhance tumorigenicity *in vivo* when compared to non-CSC or parental UM1 cells. We will also investigate if CREB-1 is required for regulating the self- renewal and/or differentiation of oral/head and neck CSC-like cells and how the CREB pathway is activated in the oral CSC-like cells. These studies will confirm that the spherogenic UM1 cells are indeed CSCs and may demonstrate potential targets of therapeutic intervention in oral cancer.

## Abbreviations

ALDH: Aldehyde dehydrogenase; bFGF: Basic fibroblast growth factor; CBP: CREB-1-binding protein; CEACAM6: Carcinoembryonic antigen-related cell adhesion molecule 6; CID: Collision-induced dissociation; CREB-1: cAMP responsive element binding protein 1; CSCs: Cancer stem-like cells; (D)MEM: (Dulbecco’s) modified Eagle’s medium; DSG3: Desmoglein 3; EGF: Epidermal growth factor; EMT: Epithelial-mesenchymal transition; GO: Gene ontology; HNSCC: Head and neck squamous cell carcinoma; LC-MS/MS: Liquid chromatography with tandem mass spectrometry; MSK-1: Mitogen- and stress-activated protein kinase; OSCC: Oral squamous cell carcinoma; PGK-1: Phosphoglycerate kinase 1; qPCR: Quantitative polymerase chain reaction; TKT: Transketolase; TMT: Tandem mass tagging.

## Competing interests

The authors declare that they have no competing interests.

## Authors’ contributions

KM, XJL, SF and SH performed the experiments and analyzed the data. KM and SH drafted the manuscript. All authors read and approved the final manuscript.

## Supplementary Material

Additional file 1: Table S1 A list of proteins with at least one TMT-labeled and confidently matched peptide. Supplemental material for online publication if needed. Click here for file
