# Supplementary material for: Quantitative proteomic analysis of sphere-forming stem-like oral cancer cells
Source: Stem Cell Res Ther. 2013 Dec 25;4(6):156. doi: 10.1186/scrt386 (PMC4056689; doi:10.1186/scrt386)
Supplement: Additional file 1: Table S1 — A list of proteins with at least one TMT-labeled and confidently matched peptide. Supplemental material for online publication if needed. [file scrt386-S1.pdf]

Supplemental table: A list of proteins with at least one TMT-labeled and confidently matched peptide.

Accession	# Peptides	MW [kDa]	calc. pI	127/126	Protein Description
IPI:IPI00382756.1	1	56.3	9.17	2.609	Isoform 2 of Pleiotropic regulator 1
IPI:IPI00410017.1	2	61.1	9.07	2.592	Isoform 2 of Polyadenylate-binding protein 1
IPI:IPI00513908.3	1	215.6	5.06	2.013	Protocadherin 15
IPI:IPI00005750.1	3	255.3	9.11	1.840	Polycystic kidney disease and receptor for egg jelly-related protein precursor
IPI:IPI00412714.3	1	42.4	8.44	1.766	Isoform 4 of Plasminogen activator inhibitor 1 RNA-binding protein
IPI:IPI00384653.1	1	11.1	3.79	1.732	Prothymosin a14
IPI:IPI00719535.1	1	5.5	4.91	1.671	ATM (Fragment)
IPI:IPI00514255.2	1	167.9	8.40	1.660	Probable phospholipid-transporting ATPase VA
IPI:IPI00398994.3	1	213.6	7.88	1.654	KIAA1305 protein
IPI:IPI00640950.1	1	6.6	9.52	1.639	7 kDa protein
IPI:IPI00374479.3	1	31.8	8.57	1.605	axonemal dynein light chain
IPI:IPI00032491.1	1	99.9	7.55	1.599	Inner nuclear membrane protein Man1
IPI:IPI00426162.1	1	31.2	8.24	1.590	Isoform C of Ras association domain-containing protein 1
IPI:IPI00386863.2	1	42.9	6.21	1.586	CDNA FLJ14838 fis, clone OVARC1001726, weakly similar to APICAL-LIKE PROTEIN
IPI:IPI00216659.1	1	19.7	5.92	1.569	Isoform 2 of RNA-binding protein 8A
IPI:IPI00295857.6	2	138.2	7.66	1.507	Coatomer subunit alpha (ER-Golgi transport Protein transport)
IPI:IPI00643791.1	1	17.8	5.49	1.487	18 kDa protein
IPI:IPI00094507.1	1	29.3	6.25	1.459	UBX domain-containing protein 4
IPI:IPI00375141.1	5	412.1	5.83	1.456	Dystrophin
IPI:IPI00440933.1	1	72.3	6.73	1.453	Isoform 2 of ADAM 9 precursor
IPI:IPI00009771.5	2	69.9	5.59	1.440	Lamin-B2
IPI:IPI00220827.4	3	4.9	5.36	1.392	Thymosin beta-10
IPI:IPI00219568.3	5	44.6	8.54	1.389	Phosphoglycerate kinase, testis specific

IPI:IPI00645588.1	1	11.2	4.97	1.387	11 kDa protein
IPI:IPI00220709.3	9	33.0	4.67	1.386	Isoform 2 of Tropomyosin beta chain
IPI:IPI00401105.1	1	13.6	10.07	1.379	PREDICTED: similar to 40S ribosomal protein S25
IPI:IPI00386330.1	1	24.0	8.88	1.372	CDNA FLJ14335 fis, clone PLACE4000411
IPI:IPI00739106.2	1	27.9	8.44	1.369	PREDICTED: similar to ribosomal protein L5 isoform 1
IPI:IPI00607659.1	1	73.5	6.07	1.363	Isoform 1 of Protein C14orf145
IPI:IPI00007049.1	1	15.8	9.55	1.353	28S ribosomal protein S18c, mitochondrial precursor
IPI:IPI00167087.1	1	111.8	5.82	1.352	COL3A1 protein
IPI:IPI00414203.2	3	81.4	5.97	1.351	Predicted testis protein
IPI:IPI00216298.5	4	11.6	4.92	1.351	Thioredoxin
IPI:IPI00289800.7	2	59.2	6.84	1.339	glucose-6-phosphate dehydrogenase
IPI:IPI00291783.3	3	168.5	6.60	1.337	Gem-associated protein 5, Gem (nuclear organelle) associated protein 5
IPI:IPI00022430.1	1	44.5	8.19	1.335	Glyceraldehyde-3-phosphate dehydrogenase, testis-specific
IPI:IPI00410096.1	3	197.5	6.13	1.333	selective LIM binding factor homolog
IPI:IPI00084828.1	1	67.5	6.96	1.331	Isoform 1 of Syntaxin-binding protein 1
IPI:IPI00215780.4	2	15.9	10.32	1.329	40S ribosomal protein S19
IPI:IPI00023211.1	1	26.0	7.94	1.323	Rho-related GTP-binding protein Rho6
IPI:IPI00375609.3	2	48.7	5.97	1.322	Chromosome 10 open reading frame 39
IPI:IPI00465315.5	3	11.6	9.57	1.314	Cytochrome c
IPI:IPI00165045.3	1	257.0	8.34	1.308	calcium channel, voltage-dependent, alpha 1E subunit
IPI:IPI00747357.1	1	7.9	10.45	1.295	8 kDa protein
IPI:IPI00643853.1	2	20.8	5.02	1.283	Chloride intracellular channel 1
IPI:IPI00183085.2	2	35.2	6.34	1.282	Highly similar to NDRG1 protein (N-myc downstream regulated 1)
IPI:IPI00414898.3	1	64.4	8.51	1.276	PREDICTED: hypothetical protein LOC23349
IPI:IPI00746441.1	1	43.6	5.26	1.274	Neutral sphingomyelinase
IPI:IPI00654746.1	1	30.2	9.14	1.273	Hypothetical protein FAM7A2
IPI:IPI00166417.6	1	30.6	6.93	1.263	CDNA FLJ90556 fis, clone OVARC1000956

IPI:IPI00005705.1	2	37.0	6.54	1.258	Serine/threonine-protein phosphatase 1, catalytic subunit, gamma isozyme
IPI:IPI00718911.1	1	10.7	10.42	1.256	Ribosomal protein L28 variant (Fragment)
IPI:IPI00442054.1	1	51.8	7.88	1.255	CDNA FLJ16524 fis, clone OCBBF2003327, moderately similar to ADAM-TS 6
IPI:IPI00016958.1	1	14.6	11.52	1.254	R33683_2
IPI:IPI00555687.1	1	24.6	6.98	1.253	Isoform 7 of Shugoshin-like 1
IPI:IPI00169383.2	11	44.5	8.10	1.247	Phosphoglycerate kinase 1
IPI:IPI00003815.2	1	23.1	5.11	1.246	Rho GDP-dissociation inhibitor 1
IPI:IPI00218110.1	1	12.1	6.51	1.239	Isocitrate dehydrogenase 3 (NAD+) gamma-like
IPI:IPI00398776.3	53	512.3	5.77	1.238	plectin 1 isoform 7 (focal adhesion)
IPI:IPI00021812.1	2	312.3	6.73	1.234	Neuroblast differentiation-associated protein AHNAK (required for cell differentiation)
IPI:IPI00658156.1	1	78.0	6.00	1.232	Isoform 4 of Serologically defined colon cancer antigen 8
IPI:IPI00513941.2	1	13.1	6.61	1.229	SAR1a gene homolog 1
IPI:IPI00004419.3	1	200.6	7.33	1.228	Zinc finger protein 646
IPI:IPI00179700.3	3	11.7	10.32	1.227	High mobility group protein HMG-I/HMG-Y
IPI:IPI00027223.2	1	46.6	7.01	1.227	Isocitrate dehydrogenase [NADP] cytoplasmic
IPI:IPI00444145.1	1	24.7	10.73	1.223	CDNA FLJ45820 fis, clone NT2RP8001407
IPI:IPI00008433.3	1	22.7	9.72	1.222	40S ribosomal protein S5
IPI:IPI00220301.4	3	24.9	6.38	1.218	Peroxiredoxin-6
IPI:IPI00243278.1	2	11.4	10.29	1.213	PREDICTED: similar to ribosomal protein L31
IPI:IPI00554618.1	1	63.7	6.10	1.210	basal cell adhesion molecule isoform 2 precursor
IPI:IPI00218353.3	3	121.0	7.75	1.210	Isoform B of Probable cation-transporting ATPase 13A1
IPI:IPI00215911.2	1	35.4	8.12	1.208	DNA-(apurinic or apyrimidinic site) lyase
IPI:IPI00384051.4	1	27.2	5.58	1.205	Proteasome activator complex subunit 2
IPI:IPI00745516.1	1	10.3	9.74	1.194	Similar to High mobility group protein HMG1
IPI:IPI00743912.1	9	74.2	4.63	1.189	Isoform 2 of Nucleolin (pre-rRNA transcription and ribosome assembly)
IPI:IPI00740650.2	2	192.3	5.40	1.187	Stereocilin-like protein

IPI:IPI00748645.1 3	19.9	10.51	1.186	Similar to 60S ribosomal protein L6
IPI:IPI00654615.1 2	18.9	9.20	1.182	Dehydrogenase/reductase SDR family member 9 precursor
IPI:IPI00394706.1 1	15.3	4.58	1.178	PHAPI protein (Fragment)
IPI:IPI00413807.3 2	77.2	8.44	1.175	Zinc finger protein 17, isoform 1 (transcriptional regulation)
IPI:IPI00382617.1 2	31.5	7.36	1.173	P37 AUF1 (Heterogeneous nuclear ribonucleoprotein D0)
IPI:IPI00449176.2 2	52.8	8.15	1.172	DENN domain-containing protein 1A (Connecdenn 1), Guanine nucleotide exchange factor
IPI:IPI00642422.1 2	300.4	9.35	1.170	Transcriptional regulatory protein Ash1
IPI:IPI00384556.1 1	75.9	8.70	1.169	Ribosomal RNA upstream binding transcription factor (Fragment)
IPI:IPI00513736.1 1	53.0	9.50	1.166	DNA methyltransferase 1-associated protein 1
IPI:IPI00012772.7 2	27.9	11.03	1.164	60S ribosomal protein L8
IPI:IPI00718994.1 1	69.0	6.32	1.163	Protein
IPI:IPI00328713.3 1	98.4	8.62	1.162	Zinc finger protein 546
IPI:IPI00748427.1 1	52.8	7.31	1.161	53 kDa protein
IPI:IPI00642560.1 3	23.2	8.51	1.156	23 kDa protein
IPI:IPI00221089.4 3	17.1	10.54	1.156	40S ribosomal protein S13
IPI:IPI00401922.1 1	27.4	5.02	1.152	27 kDa protein
IPI:IPI00220756.1 1	103.2	5.73	1.151	Isoform Alpha-7X2DA of Integrin alpha-7 precursor
IPI:IPI00428056.1 2	16.1	9.00	1.151	BBF2H7/FUS protein (nucleic acid binding)
IPI:IPI00021999.1 1	62.3	7.88	1.149	Interleukin-18 receptor 1 precursor
IPI:IPI00479701.2 1	125.7	9.22	1.143	PREDICTED: similar to transmembrane channel-like gene family 3
IPI:IPI00024295.1 1	84.7	7.52	1.142	Zinc finger X-linked protein ZXDA
IPI:IPI00396227.2 1	91.9	8.66	1.140	Isoform 4 of Regulating synaptic membrane exocytosis protein 2
IPI:IPI00260318.2 1	30.1	9.11	1.134	cyclin-dependent kinase 2 isoform 2
IPI:IPI00414922.3 2	14.5	7.21	1.133	ribosomal protein S12
IPI:IPI00410344.2 2	108.9	8.62	1.132	Serine/threonine-protein kinase PLK4
IPI:IPI00480149.2 1	24.9	7.62	1.131	25 kDa protein
IPI:IPI00759613.1 20	3651.0	6.44	1.131	Isoform 5 of Titin

IPI:IPI00513927.1	2	20.9	6.55	1.131	Glutathione S-transferase omega 1
IPI:IPI00396485.3	18	50.1	9.01	1.130	Elongation factor 1-alpha 1
IPI:IPI00021428.1	11	42.0	5.39	1.125	Actin, alpha skeletal muscle
IPI:IPI00010471.4	8	70.1	5.33	1.124	Plastin-2
IPI:IPI00012190.1	1	61.3	6.39	1.123	Putative dimethylaniline monooxygenase [N-oxide-forming] 6
IPI:IPI00743501.1	1	70.2	9.23	1.123	Hypothetical protein DKFZp686A04192 (Fragment)
IPI:IPI00007752.1	13	49.8	4.89	1.122	Tubulin beta-2C chain
IPI:IPI00166740.1	1	30.6	11.36	1.121	Hypothetical protein (Fragment)
IPI:IPI00253411.3	1	7.7	4.70	1.121	PREDICTED: similar to Tubulin alpha-4 chain
IPI:IPI00298345.1	1	67.5	7.58	1.121	Isoform 1 of Tyrosine-protein kinase RYK precursor
IPI:IPI00005154.1	1	81.0	6.87	1.117	Structure-specific recognition protein 1
IPI:IPI00455167.3	2	25.3	8.15	1.116	Dihydrodiol dehydrogenase DD2
IPI:IPI00744455.1	1	47.1	9.20	1.116	PREDICTED: formin binding protein 3 isoform 2
IPI:IPI00478277.2	2	24.5	9.91	1.115	24 kDa protein, similar to ribosomal protein L10a
IPI:IPI00292438.6	1	38.2	8.18	1.113	Protein FAM26C
IPI:IPI00514411.1	1	159.2	5.01	1.113	Isoform 2 of Hepatitis B virus X-associated protein
IPI:IPI00431747.1	2	45.6	8.48	1.112	nuclear receptor subfamily 1, group D, member 2
IPI:IPI00152780.2	1	90.3	8.28	1.112	tumor protein p53 inducible protein 5
IPI:IPI00446745.1	1	27.4	8.29	1.111	CDNA FLJ41196 fis, clone BRACE2045772
IPI:IPI00478539.2	7	32.4	8.87	1.110	Similar to Heterogeneous nuclear ribonucleoprotein A1
IPI:IPI00178440.2	1	24.6	4.67	1.107	Elongation factor 1-beta
IPI:IPI00218188.1	1	83.0	6.42	1.105	Isoform Beta of Signal transducer and activator of transcription 1-alpha/beta
IPI:IPI00644361.2	1	33.9	6.54	1.104	Arylamine N-acetyltransferase 1
IPI:IPI00217348.1	1	35.7	9.22	1.102	Isoform 2 of Opsin-3
IPI:IPI00646262.1	1	28.1	6.54	1.102	28 kDa protein
IPI:IPI00100980.9	2	61.1	6.46	1.101	EH-domain-containing protein 2
IPI:IPI00162547.2	2	139.3	7.06	1.097	Latrophilin-3 precursor (GPCR)
IPI:IPI00019869.3	3	11.1	4.78	1.097	S100A2 protein
IPI:IPI00304232.1	1	47.7	5.90	1.094	WD-repeat protein 12

IPI:IPI00646651.1	1	13.0	6.14	1.093	Rho GTPase activating protein 21
IPI:IPI00013485.3	5	31.3	10.24	1.092	40S ribosomal protein S2
IPI:IPI00216430.2	1	54.5	6.54	1.091	Isoform 2 of M-phase inducer phosphatase 1
IPI:IPI00217468.2	6	22.4	10.92	1.090	Histone H1.5
IPI:IPI00007144.1	1	17.2	10.55	1.089	60S ribosomal protein L26-like 1
IPI:IPI00328826.5	1	143.4	6.14	1.089	Isoform 2 of Calcium-dependent secretion activator 2
IPI:IPI00642325.1	1	105.0	7.24	1.088	IQ motif and Sec7 domain 2
IPI:IPI00401287.1	1	20.0	7.39	1.088	CDNA FLJ42909 fis, clone BRHIP3020182
IPI:IPI00299010.3	1	88.1	8.57	1.087	Isoform 1 of Paraplegin
IPI:IPI00030790.7	1	180.8	5.87	1.085	hypothetical protein LOC23247
IPI:IPI00640006.1	1	45.6	6.24	1.084	GDP dissociation inhibitor 2
IPI:IPI00008864.1	1	52.3	6.73	1.083	Link guanine nucleotide exchange factor II
IPI:IPI00024095.2	2	36.2	5.92	1.082	Annexin A3
IPI:IPI00640193.1	1	12.2	9.99	1.081	Hypothetical protein
IPI:IPI00741799.1	1	10.5	9.41	1.080	PREDICTED: similar to Rho-related GTP-binding protein RhoG
IPI:IPI00176903.2	1	43.4	5.60	1.079	Isoform 1 of Polymerase I and transcript release factor
IPI:IPI00297953.3	1	65.2	7.23	1.078	Hypothetical protein FLJ40311
IPI:IPI00008438.1	1	18.9	10.15	1.076	40S ribosomal protein S10
IPI:IPI00303797.3	1	84.4	7.53	1.076	B-Raf proto-oncogene serine/threonine-protein kinase
IPI:IPI00646304.3	3	23.7	9.41	1.075	peptidylprolyl isomerase B precursor
IPI:IPI00744062.1	1	121.6	8.54	1.075	transcription elongation regulator 1 isoform 2
IPI:IPI00384298.6	3	143.9	10.81	1.074	spindle assembly associated Sfi1 homolog isoform b
IPI:IPI00003084.1	1	22.3	5.17	1.073	DR1-associated protein 1
IPI:IPI00026260.1	2	17.3	8.41	1.072	Nucleoside diphosphate kinase B
IPI:IPI00748557.1	2	56.0	5.71	1.071	Serine/threonine protein phosphatase
IPI:IPI00170550.1	1	52.0	7.40	1.071	tigger transposable element derived 3
IPI:IPI00645904.1	1	44.2	7.03	1.070	44 kDa protein
IPI:IPI00646171.1	1	44.4	5.78	1.070	Hypothetical protein DKFZp434A1315
IPI:IPI00739191.1	2	53.3	5.54	1.070	PREDICTED: similar to SET protein
IPI:IPI00643920.2	10	67.8	7.66	1.070	Transketolase

IPI:IPI00296022.1	1	10.7	4.44	1.070	Ubiquinol-cytochrome c reductase complex 11 kDa protein, mitochondrial precursor
IPI:IPI00183695.8	3	11.1	7.37	1.069	Protein S100-A10
IPI:IPI00291796.2	3	199.9	6.06	1.069	BTB (POZ) domain containing 12
IPI:IPI00644576.1	29	276.4	6.05	1.069	Filamin A, alpha
IPI:IPI00018146.1	7	27.7	4.78	1.067	14-3-3 protein theta
IPI:IPI00619898.2	3	26.9	9.39	1.066	Hypothetical protein (Fragment)
IPI:IPI00025091.1	1	18.4	10.30	1.066	40S ribosomal protein S11
IPI:IPI00019472.4	1	56.6	5.48	1.065	Neutral amino acid transporter B(0)
IPI:IPI00644712.3	5	69.7	6.64	1.065	ATP-dependent DNA helicase 2 subunit 1
IPI:IPI00644196.1	7	25.7	8.57	1.064	SERPINB5 protein
IPI:IPI00333314.4	2	68.7	9.36	1.064	Zinc finger protein 85
IPI:IPI00027422.1	4	202.0	6.09	1.064	Integrin beta-4 precursor
IPI:IPI00221222.6	1	14.3	9.60	1.063	Activated RNA polymerase II transcriptional coactivator p15
IPI:IPI00012011.5	9	18.4	8.09	1.062	Cofilin-1
IPI:IPI00465164.3	1	168.2	8.05	1.060	PREDICTED: similar to SMF protein
IPI:IPI00217391.4	2	164.8	4.48	1.059	lemur tyrosine kinase 2
IPI:IPI00003035.1	1	34.7	9.04	1.058	Protein C15orf29
IPI:IPI00180983.1	2	61.8	8.25	1.057	insulin-like growth factor 2 mRNA binding protein 2 isoform b
IPI:IPI00383296.4	2	73.4	8.82	1.056	Heterogeneous nuclear ribonucleoprotein M
IPI:IPI00217030.9	1	29.4	10.15	1.055	40S ribosomal protein S4, X isoform
IPI:IPI00647495.1	1	22.2	7.01	1.055	22 kDa protein
IPI:IPI00410274.2	2	33.4	8.27	1.053	Hypothetical protein DKFZp451E0719
IPI:IPI00453473.5	6	11.2	11.36	1.052	Histone H4
IPI:IPI00550502.4	3	99.4	7.09	1.052	MCF.2 cell line derived transforming sequence-like
IPI:IPI00382781.1	1	23.6	9.41	1.050	OTTHUMP00000017281
IPI:IPI00418169.2	20	40.4	8.37	1.049	annexin A2 isoform 1
IPI:IPI00744194.1	1	16.7	5.34	1.049	Similar to Sodium/potassium-transporting ATPase alpha-1 chain precursor
IPI:IPI00219954.1	2	73.8	7.15	1.048	Isoform 2 of Exocyst complex component 3

IPI:IPI00290954.2	1	43.9	6.09	1.048	Isoform 2 of Dipeptidase 2 precursor
IPI:IPI00556157.1	3	35.5	8.98	1.047	Polypyrimidine tract-binding protein 1 isoform c variant (Fragment)
IPI:IPI00013388.2	1	4.4	8.25	1.047	Conserved hypothetical protein
IPI:IPI00027821.3	1	55.9	8.02	1.047	Trypsin inhibitor
IPI:IPI00011285.1	1	81.8	5.67	1.045	Calpain-1 catalytic subunit
IPI:IPI00642237.1	1	18.3	9.01	1.044	Nuclear factor I/B
IPI:IPI00478908.2	14	49.7	5.10	1.044	50 kDa protein
IPI:IPI00465260.3	3	84.6	7.77	1.044	GARS protein
IPI:IPI00745266.1	1	66.7	6.34	1.044	Eukaryotic translation initiation factor 3 subunit 6-interacting protein
IPI:IPI00102070.5	2	52.2	5.02	1.043	calcium binding and coiled-coil domain 2
IPI:IPI00472082.2	10	42.3	6.21	1.043	Serpin B5 precursor
IPI:IPI00399098.2	1	21.0	5.03	1.043	CDNA FLJ43276 fis, clone KIDNE2011532, moderately similar to Homo sapiens melanoma-associated chondroitin sulfate proteoglycan 4
IPI:IPI00217467.2	8	21.7	11.03	1.043	Histone H1.4
IPI:IPI00009862.4	1	51.8	5.06	1.042	Cerebellar degeneration-related protein 2
IPI:IPI00219365.2	7	67.6	6.40	1.042	Moesin
IPI:IPI00386491.5	4	88.8	5.78	1.041	Isoform Short of Heterogenous nuclear ribonucleoprotein U
IPI:IPI00029273.1	1	155.4	7.33	1.041	Hepatocyte growth factor receptor precursor
IPI:IPI00337325.2	2	84.0	5.77	1.040	Isoform A of Hyaluronan mediated motility receptor
IPI:IPI00009236.5	2	20.5	6.02	1.039	caveolin 1
IPI:IPI00556485.2	3	27.4	8.24	1.039	RPLP0 protein
IPI:IPI00005996.1	1	11.8	10.62	1.037	High mobility group protein HMGI-C
IPI:IPI00160901.3	1	76.6	9.44	1.037	G2 and S phase expressed protein 1
IPI:IPI00551024.4	1	58.9	7.49	1.037	Dihydroxyacetone kinase
IPI:IPI00382470.2	10	98.1	5.17	1.033	Heat shock protein HSP 90-alpha 2
IPI:IPI00646059.1	7	31.1	4.18	1.033	SET translocation
IPI:IPI00749513.1	1	115.6	6.05	1.032	polycystic kidney disease 1-like isoform a

IPI:IPI00160395.3	3	35.9	5.41	1.032	Homer-3B protein
IPI:IPI00304648.2	1	88.3	6.11	1.031	MLCK protein
IPI:IPI00446355.1	1	18.4	7.56	1.027	CDNA FLJ41803 fis, clone NHNPC2002749
IPI:IPI00742692.1	2	11.5	6.68	1.025	Actin-like protein (Fragment)
IPI:IPI00745153.1	3	113.1	7.94	1.025	Isoform 4 of Regulator of telomere elongation helicase 1
IPI:IPI00219833.2	1	18.3	9.03	1.024	Mitochondrial import inner membrane translocase subunit Tim17-B
IPI:IPI00740535.1	1	28.0	10.59	1.023	PREDICTED: similar to Kinesin heavy chain isoform 5C
IPI:IPI00219153.3	2	14.6	9.19	1.022	60S ribosomal protein L22
IPI:IPI00745897.1	1	30.8	5.45	1.021	Similar to Rabring 7
IPI:IPI00329801.1	9	35.8	5.05	1.021	Annexin A5
IPI:IPI00302112.1	2	51.8	9.22	1.021	Isoform B of Dual specificity mitogen-activated protein kinase kinase 7
IPI:IPI00429684.1	1	19.2	9.06	1.020	Fibrosin-1
IPI:IPI00216464.9	3	73.0	6.16	1.019	Hypothetical protein DKFZp761L2322
IPI:IPI00021145.1	3	66.5	9.10	1.019	Isoform 1 of Dual specificity protein phosphatase CDC14A
IPI:IPI00442874.1	1	18.9	10.68	1.018	Hypothetical protein FLJ26465
IPI:IPI00220967.1	5	152.1	8.66	1.018	Isoform 1 of Ribosome-binding protein 1
IPI:IPI00010414.3	1	35.9	7.02	1.017	PDZ and LIM domain protein 1
IPI:IPI00004656.1	1	13.7	6.52	1.017	Beta-2-microglobulin precursor
IPI:IPI00374179.2	1	20.0	8.40	1.017	Full-length cDNA clone CS0DJ006YA22 of T cells (Fragment)
IPI:IPI00010156.1	1	62.6	6.49	1.017	Mitogen-activated protein kinase 4
IPI:IPI00747425.1	1	12.4	11.46	1.017	Similar to ATR-interacting protein
IPI:IPI00043862.4	1	78.9	9.35	1.016	IQCA protein
IPI:IPI00025491.1	6	46.1	5.48	1.016	Eukaryotic initiation factor 4A-I
IPI:IPI00386368.1	1	20.6	9.76	1.015	Seven transmembrane helix receptor
IPI:IPI00396321.1	1	34.9	9.57	1.015	Leucine-rich repeat-containing protein 59
IPI:IPI00217465.4	7	21.2	10.93	1.014	Histone H1.2
IPI:IPI00023407.3	2	128.1	6.92	1.014	Nck-associated protein 1-like
IPI:IPI00178721.2	1	44.8	8.24	1.013	Putative SB115 protein

IPI:IPI00004362.2	1	112.8	7.85	1.013	MORC family CW-type zinc finger 1
IPI:IPI00550558.5	1	75.2	6.83	1.012	Protein O-linked-mannose beta-1,2-N-acetylglucosaminyltransferase 1
IPI:IPI00075248.1(	5	16.7	4.22	1.012	Calmodulin
IPI:IPI00219217.2	1	36.5	6.05	1.012	L-lactate dehydrogenase B chain
IPI:IPI00297058.2	1	34.3	7.81	1.011	Hypothetical protein FLJ23235
IPI:IPI00297224.4	2	89.3	6.43	1.011	PREDICTED: hypothetical protein LOC26032
IPI:IPI00307665.3	1	150.9	9.14	1.010	zinc finger protein 518
IPI:IPI00001639.2	1	97.1	4.78	1.009	Importin beta-1 subunit
IPI:IPI00437751.1	1	149.6	6.39	1.009	Angiotensin-converting enzyme, somatic isoform precursor
IPI:IPI00297671.3	1	115.5	9.54	1.008	Cyclin-dependent kinase-like 5
IPI:IPI00604407.1	1	9.7	4.75	1.007	RNA binding motif protein 3 isoform b
IPI:IPI00479359.6	6	69.4	6.27	1.007	villin 2
IPI:IPI00163187.9	3	54.4	7.24	1.007	Fascin
IPI:IPI00736001.1	1	18.2	9.31	1.006	PREDICTED: similar to zinc finger protein 114
IPI:IPI00607708.1	5	36.3	7.77	1.006	Isoform 2 of L-lactate dehydrogenase A chain
IPI:IPI00298393.3	1	38.5	9.66	1.004	CDNA FLJ38738 fis, clone KIDNE2011508, highly similar to Homo sapiens hNBL4
IPI:IPI00647226.2	1	20.0	5.10	1.003	PREDICTED: similar to ADP-ribosylation factor 1 like
IPI:IPI00009151.3	1	48.7	7.33	1.003	Isoform 2 of Butyrophilin-like protein 3 precursor
IPI:IPI00747629.1	7	28.8	4.81	1.002	Isoform 3 of Tropomyosin alpha-3 chain
IPI:IPI00641823.1	1	27.5	6.29	1.000	OLFML2B protein
IPI:IPI00005634.3	2	175.4	7.53	0.999	Hypothetical protein KIAA0372
IPI:IPI00556655.1	2	41.8	7.87	0.997	LAMP1 protein variant (Fragment)
IPI:IPI00221235.3	1	162.0	5.50	0.997	nucleoporin 160kDa
IPI:IPI00007117.1	2	46.6	5.63	0.997	Plasminogen activator inhibitor 2 precursor
IPI:IPI00062884.3	1	57.4	5.33	0.997	cortactin isoform b
IPI:IPI00411979.2	2	107.0	7.05	0.997	formin-like 3 isoform 1
IPI:IPI00719202.1	2	44.3	7.02	0.996	MHC class I antigen heavy chain
IPI:IPI00003881.5	2	45.6	5.58	0.996	heterogeneous nuclear ribonucleoprotein F

IPI:IPI00645201.1	3	21.9	10.36	0.996	Ribosomal protein S8
IPI:IPI00220667.2	2	101.0	7.05	0.993	Isoform 4 of Hexokinase-1
IPI:IPI00741181.1	1	8.2	6.35	0.993	PREDICTED: similar to Heat shock protein HSP 90-beta
IPI:IPI00021347.1	2	17.9	8.51	0.993	Ubiquitin-conjugating enzyme E2 L3
IPI:IPI00011654.2	14	49.6	4.89	0.992	Tubulin beta-2 chain
IPI:IPI00719047.1	2	21.7	10.07	0.992	Hypothetical protein
IPI:IPI00152527.3	1	124.1	6.52	0.991	DNA helicase HEL308
IPI:IPI00010162.1	1	35.9	9.64	0.991	Isoform 2 of Brain mitochondrial carrier protein 1
IPI:IPI00744721.1	2	49.6	9.70	0.990	ALS2CR11 protein (Fragment)
IPI:IPI00746169.1	1	7.4	8.22	0.990	7 kDa protein
IPI:IPI00216694.3	8	70.8	5.60	0.990	plastin 3
IPI:IPI00736092.1	1	46.8	6.64	0.989	PREDICTED: similar to Ubiquitin carboxyl-terminal hydrolase 24
IPI:IPI00000581.5	3	31.4	5.00	0.989	Hypothetical protein DKFZp564E242
IPI:IPI00221091.8	2	14.7	10.13	0.989	40S ribosomal protein S15a
IPI:IPI00640741.1	4	19.0	6.92	0.989	19 kDa protein
IPI:IPI00644531.1	5	21.1	7.81	0.989	21 kDa protein
IPI:IPI00307660.4	1	223.7	6.62	0.988	Phospholipase C, epsilon 1
IPI:IPI00013508.5	26	103.0	5.41	0.986	Alpha-actinin-1
IPI:IPI00651715.2	1	26.8	11.44	0.985	CHST13 protein
IPI:IPI00025084.3	1	28.3	5.20	0.985	Calpain small subunit 1
IPI:IPI00642777.1	3	85.9	5.81	0.985	86 kDa protein
IPI:IPI00306332.4	2	17.8	11.25	0.984	60S ribosomal protein L24
IPI:IPI00644541.1	2	30.2	9.00	0.984	30 kDa protein
IPI:IPI00413242.2	1	84.1	8.76	0.983	CDNA FLJ37119 fis, clone BRACE2022333
IPI:IPI00759832.1	11	27.8	4.83	0.983	Isoform Short of 14-3-3 protein beta/alpha
IPI:IPI00008527.3	2	11.5	4.32	0.983	60S acidic ribosomal protein P1
IPI:IPI00465248.5	19	47.1	7.39	0.983	enolase 1
IPI:IPI00382700.1	7	271.3	5.74	0.982	Isoform 6 of Filamin-B
IPI:IPI00027157.1	5	367.4	5.06	0.982	CENP-F kinetochore protein

IPI:IPI00432707.2	1	39.1	6.13	0.982	CASP12P1
IPI:IPI00556231.1	1	24.6	7.78	0.981	Hypothetical protein
IPI:IPI00384803.1	1	15.9	5.08	0.981	CDNA FLJ38411 fis, clone FEBRA2009352
IPI:IPI00216223.1	3	119.0	7.27	0.981	Isoform Alpha-6X2A of Integrin alpha-6 precursor
IPI:IPI00745661.1	1	13.0	4.63	0.979	Conserved hypothetical protein
IPI:IPI00302927.5	1	57.8	7.83	0.979	T-complex protein 1 subunit delta
IPI:IPI00301475.5	1	46.1	10.01	0.979	Galactose-3-O-sulfotransferase 2
IPI:IPI00163646.1	3	307.4	5.39	0.978	Protein kinase A anchoring protein Ht31
IPI:IPI00306984.1	1	123.8	6.98	0.978	Potassium voltage-gated channel subfamily H member 8
IPI:IPI00220740.1	7	29.4	4.61	0.977	Isoform 2 of Nucleophosmin
IPI:IPI00018871.1	1	21.5	8.43	0.977	ADP-ribosylation factor-like protein 8B
IPI:IPI00470692.1	2	92.9	6.79	0.977	Isoform 1 of Netrin receptor UNC5A precursor
IPI:IPI00167796.1	1	17.2	7.71	0.977	Protein C14orf65
IPI:IPI00021439.1	19	41.7	5.48	0.977	Actin, cytoplasmic 1
IPI:IPI00171716.3	2	270.7	6.89	0.976	OTTHUMP00000028696
IPI:IPI00419958.4	2	79.4	5.64	0.975	CASC1 protein
IPI:IPI00385055.3	2	100.4	5.96	0.975	catenin, alpha 2
IPI:IPI00003091.1	2	125.8	5.02	0.975	Isoform 1 of Protein phosphatase 1 regulatory subunit 3A
IPI:IPI00000737.3	1	35.4	6.06	0.975	Tetraspanin-12
IPI:IPI00298547.3	4	19.9	6.79	0.974	Protein DJ-1
IPI:IPI00009659.3	3	36.9	5.97	0.974	Protein C20orf77
IPI:IPI00028078.1	1	59.7	8.73	0.974	Isoform A of Potassium channel subfamily K member 10
IPI:IPI00219486.1	1	15.1	10.89	0.974	Isoform 2 of 40S ribosomal protein S24
IPI:IPI00640033.1	1	12.2	10.05	0.973	Hypothetical protein
IPI:IPI00414696.1	7	36.0	8.65	0.972	Isoform A2 of Heterogeneous nuclear ribonucleoproteins A2/B1
IPI:IPI00647334.1	1	39.1	6.60	0.972	SETMAR protein
IPI:IPI00749373.1	1	8.1	11.12	0.971	8 kDa protein
IPI:IPI00220766.3	1	20.6	5.47	0.971	Lactoylglutathione lyase
IPI:IPI00418823.3	1	29.4	8.18	0.971	PREDICTED: similar to lactate dehydrogenase A-like 6B
IPI:IPI00013894.1	1	62.6	6.80	0.969	Stress-induced-phosphoprotein 1

IPI:IPI00514386.1	1	80.2	6.23	0.968	Novel protein
IPI:IPI00028116.1	1	24.5	8.62	0.968	ER lumen protein retaining receptor 1
IPI:IPI00398106.1	1	15.4	11.52	0.968	PREDICTED: similar to HIStone family member
IPI:IPI00001591.1	1	14.5	8.35	0.968	Apoptosis related protein APR-5 (Fragment)
IPI:IPI00003918.5	5	47.5	11.06	0.966	60S ribosomal protein L4
IPI:IPI00219058.1	1	69.0	6.42	0.966	Isoform 2 of Lethal(3)malignant brain tumor-like 2 protein
IPI:IPI00218209.1	1	41.4	8.25	0.965	Isoform Short of B2 bradykinin receptor
IPI:IPI00010182.3	2	10.0	6.57	0.965	DBI protein
IPI:IPI00055753.6	1	43.8	9.66	0.965	Methionyl-tRNA formyltransferase, mitochondrial precursor
IPI:IPI00000839.1	3	101.8	8.82	0.965	Metabotropic glutamate receptor 4 precursor
IPI:IPI00738152.1	2	22.1	11.66	0.964	PREDICTED: similar to ribosomal protein L15 isoform 2
IPI:IPI00220119.4	1	117.3	5.55	0.963	Isoform 1 of SWI/SNF-related matrix-associated actin-dependent regulator of chromatin subfamily A containing DEAD/H box 1
IPI:IPI00383754.1	1	12.1	4.72	0.963	Tryptophanyl-tRNA synthetase (Fragment)
IPI:IPI00657857.1	1	21.7	8.02	0.963	22 kDa protein
IPI:IPI00045396.1	1	37.1	4.59	0.962	Isoform 2 of Calumenin precursor
IPI:IPI00736358.1	2	448.4	7.17	0.962	PREDICTED: similar to protein kinase, DNA-activated, catalytic polypeptide
IPI:IPI00746091.1	1	12.8	6.57	0.961	motilin isoform 2 preproprotein
IPI:IPI00221178.1	1	19.9	5.48	0.961	Isoform 2 of Tumor protein D54
IPI:IPI00514330.5	1	25.5	4.55	0.961	Hepatoma-derived growth factor
IPI:IPI00186290.5	9	95.1	6.83	0.960	Elongation factor 2
IPI:IPI00218823.3	1	293.5	8.22	0.959	Isoform 1 of Myeloid/lymphoid or mixed-lineage leukemia protein 4
IPI:IPI00217826.1	2	46.4	6.52	0.959	Leucine-rich repeat-containing protein 34
IPI:IPI00747119.1	2	127.1	8.95	0.958	plakophilin 4 isoform b
IPI:IPI00639819.1	1	33.4	6.55	0.957	TAR DNA binding protein
IPI:IPI00556258.1	1	29.1	6.99	0.957	Aldo-keto reductase family 1, member B1 variant (Fragment)
IPI:IPI00180730.1	9	50.1	8.95	0.957	Similar to Elongation factor 1-alpha 1
IPI:IPI00065351.3	2	70.5	9.50	0.957	Hypothetical protein FLJ25008

IPI:IPI00030268.4	2	89.1	4.21	0.956	Protein C14orf155
IPI:IPI00736329.1	2	410.8	5.40	0.956	PREDICTED: piccolo isoform 4
IPI:IPI00376890.5	1	43.8	9.99	0.955	44 kDa protein
IPI:IPI00302990.3	1	42.1	4.94	0.955	WD-repeat protein 55
IPI:IPI00006451.5	3	82.6	6.81	0.954	Vesicle-fusing ATPase
IPI:IPI00027493.1	7	57.9	5.35	0.954	4F2 cell-surface antigen heavy chain
IPI:IPI00166002.3	1	60.1	8.73	0.953	PSD3 protein
IPI:IPI00219950.2	3	23.7	9.11	0.953	Isoform 2 of Proteasome subunit alpha type 7-like
IPI:IPI00465048.5	3	75.0	6.51	0.953	RUN and FYVE domain-containing 2
IPI:IPI00739762.1	3	37.1	8.25	0.952	PREDICTED: similar to Elongation factor 1-gamma (EF-1-gamma) (eEF-1B gamma) isoform 8
IPI:IPI00003515.1	3	227.5	5.27	0.952	Thyroid receptor-interacting protein 11
IPI:IPI00013895.1	6	11.7	7.12	0.952	Protein S100-A11
IPI:IPI00749001.1	1	58.5	9.77	0.951	CDNA FLJ13840 fis, clone THYRO1000783
IPI:IPI00027497.4	3	63.0	8.32	0.951	Glucose-6-phosphate isomerase
IPI:IPI00747194.1	2	15.5	8.53	0.950	15 kDa protein
IPI:IPI00029631.1	1	12.3	5.92	0.950	Enhancer of rudimentary homolog
IPI:IPI00552483.1	1	53.1	9.09	0.950	U2 (RNU2) small nuclear RNA auxiliary factor 2 isoform b
IPI:IPI00641961.1	2	315.7	5.34	0.949	Collagen, type XII, alpha 1
IPI:IPI00401193.3	1	50.9	5.36	0.949	KIAA1147 protein (Fragment)
IPI:IPI00219622.2	1	25.8	7.43	0.948	Proteasome subunit alpha type 2
IPI:IPI00306933.1	4	229.2	8.51	0.948	Isoform Short of Myosin-9B
IPI:IPI00220642.6	8	28.2	4.89	0.947	14-3-3 protein gamma
IPI:IPI00329556.6	3	191.9	5.99	0.947	hypothetical protein LOC80217
IPI:IPI00292858.4	1	49.9	5.53	0.947	Thymidine phosphorylase precursor
IPI:IPI00027463.1	6	10.2	5.48	0.946	Protein S100-A6
IPI:IPI00220552.1	1	93.1	6.43	0.945	Isoform 2 of Short transient receptor potential channel 6
IPI:IPI00221092.7	2	16.3	10.21	0.944	40S ribosomal protein S16
IPI:IPI00639893.1	2	76.0	5.64	0.944	76 kDa protein
IPI:IPI00008569.1	1	22.4	6.92	0.944	YKT6 v-SNARE protein

IPI:IPI00032633.4	2	50.8	7.30	0.944	Ubiquinone biosynthesis monooxgenase COQ6
IPI:IPI00166767.3	1	58.6	9.29	0.944	Isoform 1 of F-box/LRR-repeat protein 6
IPI:IPI00219757.1[illegible]	12	23.2	5.64	0.944	Glutathione S-transferase P
IPI:IPI00640338.1	2	25.2	6.23	0.942	25 kDa protein
IPI:IPI00017964.1	1	13.9	10.32	0.941	Small nuclear ribonucleoprotein Sm D3
IPI:IPI00479186.4	25	57.9	7.84	0.940	pyruvate kinase 3 isoform 1
IPI:IPI00003377.1	2	27.4	11.82	0.940	Isoform 1 of Splicing factor, arginine/serine-rich 7
IPI:IPI00457093.2	1	14.2	7.14	0.940	PREDICTED: similar to peptidylprolyl isomerase A isoform 1
IPI:IPI00397484.3	1	18.0	9.01	0.939	Zinc finger CCHC domain-containing protein 13
IPI:IPI00009342.1	9	189.1	6.48	0.938	Ras GTPase-activating-like protein IQGAP1
IPI:IPI00748761.1	1	154.8	5.67	0.938	Protein kinase PKN/PRK1, effector domain containing protein
IPI:IPI00642308.1	1	151.9	6.79	0.937	152 kDa protein
IPI:IPI00640929.1	1	10.4	5.48	0.936	10 kDa protein
IPI:IPI00024603.8	1	51.7	8.21	0.936	bruno-like 4, RNA binding protein
IPI:IPI00643014.1	2	573.5	6.04	0.936	retinoblastoma-associated factor 600
IPI:IPI00010720.1	1	59.6	5.66	0.936	T-complex protein 1 subunit epsilon
IPI:IPI00293305.1	1	87.4	5.53	0.934	Isoform Beta-1B of Integrin beta-1 precursor
IPI:IPI00297289.5	3	146.6	9.22	0.933	Ubiquitin-specific proteinase 31
IPI:IPI00215943.1	51	517.7	5.73	0.932	Isoform 3 of Plectin-1
IPI:IPI00305383.1	5	48.4	8.63	0.931	Ubiquinol-cytochrome-c reductase complex core protein 2, mitochondrial precursor
IPI:IPI00221093.6	1	15.4	9.85	0.931	40S ribosomal protein S17
IPI:IPI00007765.5	6	73.6	6.16	0.930	Stress-70 protein, mitochondrial precursor
IPI:IPI00063408.6	2	103.0	6.93	0.929	dehydrogenase E1 and transketolase domain containing protein 1
IPI:IPI00642681.1	1	6.3	10.18	0.929	6 kDa protein
IPI:IPI00470433.1	1	30.1	9.32	0.927	Multiple myeloma SET domain containing protein type III
IPI:IPI00647859.1	1	39.0	5.03	0.927	Similar to Aminopeptidase B
IPI:IPI00743093.1	2	6.4	9.50	0.927	Similar to Transcription repressor
IPI:IPI00386903.2	1	23.8	9.82	0.926	Isoform 2 of NUAK family, SNF1-like kinase 1
IPI:IPI00238077.4	1	93.1	8.54	0.924	SH3 domain containing ring finger 1

IPI:IPI00742941.1	1	13.1	12.09	0.924	13 kDa protein
IPI:IPI00014850.4	1	15.0	5.02	0.924	Astrocytic phosphoprotein PEA-15
IPI:IPI00646801.1	1	15.8	7.43	0.923	Protein-O-mannosyltransferase 1
IPI:IPI00174391.2	1	115.1	8.91	0.922	PREDICTED: similar to chromosome 9 open reading frame 36
IPI:IPI00216691.4	6	14.9	8.27	0.922	Profilin-1
IPI:IPI00025366.4	1	51.7	8.32	0.922	Citrate synthase, mitochondrial precursor
IPI:IPI00554497.1	2	160.7	7.02	0.921	Isoform 3 of Nance-Horan syndrome protein
IPI:IPI00374151.1	1	25.8	7.46	0.921	peroxiredoxin 3 isoform b
IPI:IPI00414750.2	1	27.7	8.29	0.921	Keratin-associated protein 24-1
IPI:IPI00419098.1	1	14.2	8.65	0.920	RPL12 protein
IPI:IPI00514561.1	3	47.5	5.63	0.920	Heterogeneous nuclear ribonucleoprotein K
IPI:IPI00640197.1	2	139.7	6.52	0.918	Tripeptidyl peptidase II
IPI:IPI00553164.3	2	32.7	4.87	0.918	40S ribosomal protein SA
IPI:IPI00455757.1	1	13.8	10.78	0.918	PREDICTED: similar to 60S ribosomal protein L35 isoform 1
IPI:IPI00552590.1	2	53.3	7.27	0.918	chaperonin containing TCP1, subunit 6A isoform b
IPI:IPI00552715.1	1	56.4	6.49	0.917	chaperonin containing TCP1, subunit 3 isoform c
IPI:IPI00412224.2	1	136.6	6.92	0.916	Bromodomain and WD-repeat domain-containing protein 2
IPI:IPI00297702.2	1	45.6	8.19	0.916	LOC51136 protein
IPI:IPI00023258.4	1	118.3	9.38	0.916	PREDICTED: TAF4b RNA polymerase II, TATA box binding protein (TBP)-associated factor, 105kDa
IPI:IPI00738829.1	3	238.0	4.64	0.916	PREDICTED: similar to retinitis pigmentosa 1-like 1
IPI:IPI00003865.1	19	70.9	5.52	0.914	Isoform 1 of Heat shock cognate 71 kDa protein
IPI:IPI00736509.1	3	21.3	10.49	0.914	PREDICTED: similar to 60S ribosomal protein L7 isoform 2
IPI:IPI00218474.4	6	46.8	7.71	0.913	Beta-enolase
IPI:IPI00740800.1	3	25.9	6.32	0.913	Similar to Phosphoglycerate mutase 1
IPI:IPI00642700.1	1	15.8	5.78	0.912	OTTHUMP00000030685
IPI:IPI00514669.1	1	9.4	9.36	0.912	SH3 domain binding glutamic acid-rich protein like 3
IPI:IPI00028006.1	2	22.8	7.02	0.911	Proteasome subunit beta type 2
IPI:IPI00746974.1	1	8.4	9.61	0.910	PEA15 protein
IPI:IPI00472421.1	1	113.1	5.12	0.909	serine/threonine kinase 31 isoform b

IPI:IPI00657759.1	1	95.0	7.24	0.909	Protein
IPI:IPI00465256.3	1	25.4	9.16	0.908	GTP:AMP phosphotransferase mitochondrial
IPI:IPI00008274.5	7	51.5	8.02	0.907	Adenylyl cyclase-associated protein 1
IPI:IPI00641337.2	1	40.3	8.00	0.907	Leukocyte immunoglobulin-like receptor A3
IPI:IPI00176482.6	2	131.1	8.34	0.907	highly similar to Human G2 protein
IPI:IPI00411704.8	3	16.7	5.24	0.906	Eukaryotic translation initiation factor 5A-1
IPI:IPI00031562.2	7	14.0	11.05	0.906	Histone H2A type 3
IPI:IPI00290928.2	1	44.0	8.00	0.905	Guanine nucleotide-binding protein alpha-13 subunit
IPI:IPI00647746.1	1	34.0	9.74	0.904	OTTHUMP00000021341
IPI:IPI00172421.6	1	17.9	4.48	0.904	Isoform 2 of S-phase kinase-associated protein 1A
IPI:IPI00174847.5	1	40.8	7.59	0.903	Putative glycosyltransferase
IPI:IPI00744735.1	2	141.6	8.06	0.903	IMP dehydrogenase/GMP reductase family protein
IPI:IPI00002547.1	1	74.5	7.05	0.903	Calpain-6
IPI:IPI00555576.1	1	144.6	7.03	0.902	145 kDa protein
IPI:IPI00550069.2	1	49.8	4.82	0.901	Ribonuclease inhibitor
IPI:IPI00026781.2	3	273.2	6.43	0.901	Fatty acid synthase
IPI:IPI00644989.1	3	48.1	5.08	0.900	Protein disulfide-isomerase A6 precursor
IPI:IPI00293665.6	26	59.8	8.00	0.900	Keratin, type II cytoskeletal 6B
IPI:IPI00293616.3	1	73.1	7.55	0.900	ATP-dependent RNA helicase DDX3Y
IPI:IPI00008475.1	3	57.3	5.41	0.898	Hydroxymethylglutaryl-CoA synthase, cytoplasmic
IPI:IPI00166974.3	1	36.6	6.04	0.898	aryl hydrocarbon receptor interacting protein-like 1 isoform 2
IPI:IPI00744370.1	2	26.4	5.16	0.898	Proline/glutamine-rich splicing factor
IPI:IPI00007193.5	3	196.3	5.72	0.897	Ankyrin repeat domain-containing protein 26
IPI:IPI00256795.1	1	46.4	6.30	0.897	cation channel, sperm associated 3
IPI:IPI00219219.2	1	14.6	5.50	0.897	Galectin-1
IPI:IPI00550900.1	2	19.6	4.93	0.896	Translationally-controlled tumor protein
IPI:IPI00400963.2	1	109.1	8.35	0.896	CDNA FLJ44101 fis, clone TESTI4043947
IPI:IPI00555640.1	1	50.4	7.12	0.894	Mitogen activated protein kinase 7 transcript variant 5 variant (Fragment)
IPI:IPI00412601.3	1	67.8	5.15	0.894	Isoform 2D of Cytoplasmic dynein 1 intermediate chain 2

IPI:IPI00401191.3	2	146.0	9.19	0.892	Ubinuclein-2
IPI:IPI00550232.1	1	379.0	6.39	0.891	CMYA3
IPI:IPI00643741.2	1	323.8	6.92	0.891	Isoform 2 of Chromodomain-helicase-DNA-binding protein 9
IPI:IPI00420049.2	1	71.9	7.30	0.891	PREDICTED: hypothetical protein LOC84808
IPI:IPI00739497.1	1	93.4	7.24	0.891	PREDICTED: hypothetical protein LOC79834 isoform 2
IPI:IPI00743902.1	1	7.2	8.72	0.890	Similar to Laminin alpha-2 chain precursor
IPI:IPI00465439.4	9	39.3	8.09	0.890	Fructose-bisphosphate aldolase A
IPI:IPI00746370.1	2	56.7	8.82	0.888	Synapsin II
IPI:IPI00019502.2	37	226.3	5.60	0.888	Myosin-9
IPI:IPI00759657.1	1	80.8	6.07	0.888	phospholipase A2-activating protein isoform 2
IPI:IPI00643490.1	1	59.2	5.63	0.887	Hypothetical protein DKFZp779G2333
IPI:IPI00215719.5	1	21.5	11.72	0.887	60S ribosomal protein L18
IPI:IPI00414964.2	1	12.5	9.92	0.887	12 kDa protein
IPI:IPI00027485.3	1	25.1	6.15	0.886	Eukaryotic translation initiation factor 4E
IPI:IPI00004358.3	4	96.5	6.86	0.886	Glycogen phosphorylase, brain form
IPI:IPI00549357.2	1	53.8	7.78	0.886	FAD-dependent oxidoreductase domain containing 1
IPI:IPI00479296.1	3	179.1	7.18	0.885	ATP-binding cassette, sub-family A member 8
IPI:IPI00444537.1	1	14.3	11.31	0.885	CDNA FLJ45398 fis, clone BRHIP3027947
IPI:IPI00449669.2	2	29.4	4.61	0.885	Isoform 2 of Translocon-associated protein alpha subunit precursor (ER membrane)
IPI:IPI00183572.3	3	174.0	7.02	0.884	Dedicator of cytokinesis protein 7, guanine nucleotide exchange factor
IPI:IPI00030915.1	1	127.4	8.51	0.884	Ubiquitin carboxyl-terminal hydrolase 8
IPI:IPI00383449.2	3	23.5	9.95	0.884	Ras-related protein Rab-15
IPI:IPI00017339.1	1	44.4	8.56	0.883	Splicing factor 3B subunit 4
IPI:IPI00748411.1	2	52.9	9.31	0.882	Serine hydroxymethyltransferase (interconversion of serine and glycine)
IPI:IPI00219206.1	1	20.1	5.48	0.882	Isoform 2 of Receptor tyrosine-protein kinase erbB-3 precursor
IPI:IPI00218060.1	1	34.1	5.47	0.882	MRDS1 protein (Fragment)
IPI:IPI00024674.1	1	44.3	8.07	0.881	Dual specificity mitogen-activated protein kinase kinase 4

IPI:IPI00748243.1	1	5.3	9.10	0.881	Similar to PinX1
IPI:IPI00218845.3	1	133.1	7.27	0.880	Nitric-oxide synthase, endothelial
IPI:IPI00641950.3	3	37.9	8.24	0.879	Lung cancer oncogene 7
IPI:IPI00256376.5	2	32.6	8.65	0.879	Enoyl Coenzyme A hydratase domain containing 3 variant
IPI:IPI00019326.1	2	19.4	5.83	0.878	Adrenodoxin, mitochondrial precursor
IPI:IPI00298731.2	2	99.0	9.17	0.878	Serine/threonine-protein phosphatase 1 regulatory subunit 10
IPI:IPI00220339.2	1	127.0	8.07	0.877	Similar to Nitric oxide synthase, inducible
IPI:IPI00749490.1	1	10.0	6.51	0.877	Similar to Smage-3 protein
IPI:IPI00719622.1	1	7.8	10.70	0.877	40S ribosomal protein S28
IPI:IPI00010951.1	6	552.8	5.59	0.877	Epiplakin (450 kDa epidermal antigen)
IPI:IPI00643041.2	1	24.3	7.49	0.877	GTP-binding nuclear protein Ran
IPI:IPI00744741.1	1	8.3	9.20	0.876	Similar to Ribosomal protein
IPI:IPI00479418.2	1	29.2	10.43	0.876	PREDICTED: similar to 60S ribosomal protein L7a
IPI:IPI00465028.6	12	30.5	5.71	0.875	Triosephosphate isomerase
IPI:IPI00029623.1	2	27.4	6.76	0.875	Proteasome subunit alpha type 6
IPI:IPI00000877.1	2	111.3	5.22	0.875	150 kDa oxygen-regulated protein precursor
IPI:IPI00455383.3	14	187.6	5.69	0.875	Isoform 2 of Clathrin heavy chain 1
IPI:IPI00216684.1	1	48.8	7.55	0.874	Isoform 4 of M-phase inducer phosphatase 3
IPI:IPI00413922.3	7	16.8	4.55	0.874	Isoform Smooth muscle of Myosin light polypeptide 6
IPI:IPI00161614.3	2	162.9	6.01	0.874	A-kinase anchor protein 11 isoform 2
IPI:IPI00645078.1	4	117.8	5.76	0.873	Ubiquitin-activating enzyme E1
IPI:IPI00240345.3	1	51.6	6.35	0.873	C-type lectin domain family 14 member A precursor
IPI:IPI00027230.3	5	92.4	4.84	0.873	Endoplasmin precursor (Tumor rejection antigen 1)
IPI:IPI00216484.4	1	75.6	5.49	0.873	Hypothetical protein ARMC9
IPI:IPI00409719.1	1	66.1	8.81	0.872	Isoform 2 of 72 kDa inositol polyphosphate 5-phosphatase
IPI:IPI00513933.1	1	14.7	9.23	0.872	Novel protein
IPI:IPI00217692.4	1	81.5	5.16	0.872	PCDH21 protein
IPI:IPI00103335.1	2	109.6	7.33	0.870	Glutamate receptor ionotropic, kainate 5
IPI:IPI00741244.1	1	64.1	5.73	0.869	PREDICTED: hypothetical protein LOC90379 isoform 2
IPI:IPI00744148.1	4	39.0	9.83	0.869	Isoform 1 of Core histone macro-H2A.1

IPI:IPI00013415.1	3	22.1	10.10	0.868	40S ribosomal protein S7
IPI:IPI00640400.2	1	66.3	8.65	0.868	carnitine palmitoyltransferase 1B isoform b
IPI:IPI00027003.1	1	57.0	8.51	0.868	Probable G-protein coupled receptor 176
IPI:IPI00647241.1	1	74.5	9.06	0.868	PREDICTED: similar to Zinc finger protein 443 (Kruppel-type zinc finger protein ZK1) isoform 1
IPI:IPI00759596.1	4	27.8	4.69	0.868	Heterogeneous nuclear ribonucleoproteins C1/C2
IPI:IPI00337802.3	3	100.9	7.71	0.867	Disheveled-associated activator of morphogenesis 1
IPI:IPI00007188.4	7	32.7	9.74	0.866	ADP/ATP translocase 2
IPI:IPI00719280.1	8	25.7	7.43	0.866	ubiquitin B precursor
IPI:IPI00013916.1	1	34.8	7.65	0.866	Olfactory receptor 1F1
IPI:IPI00174082.1	1	26.6	5.03	0.866	Conserved hypothetical protein
IPI:IPI00301480.5	1	134.4	6.42	0.865	1-phosphatidylinositol-4,5-bisphosphate phosphodiesterase beta 2
IPI:IPI00645085.2	1	14.8	6.92	0.864	PREDICTED: similar to Von Ebners gland protein precursor
IPI:IPI00025874.2	4	72.7	6.28	0.864	Dolichyl-diphosphooligosaccharide-protein glycosyltransferase 67 kDa subunit precursor (Ribophorin I)
IPI:IPI00550239.3	4	20.7	10.84	0.863	Histone H1.0
IPI:IPI00020599.1	7	48.1	4.44	0.862	Calreticulin precursor
IPI:IPI00022977.1	1	42.6	5.59	0.862	Creatine kinase B-type
IPI:IPI00028723.1	1	16.4	5.30	0.862	Isoform 2 of Ganglioside-induced differentiation-associated protein 1-like 1
IPI:IPI00012503.1	2	58.1	5.17	0.861	Isoform Sap-mu-0 of Proactivator polypeptide precursor
IPI:IPI00027442.3	2	106.7	5.49	0.861	Alanyl-tRNA synthetase
IPI:IPI00002821.3	2	23.1	10.93	0.860	60S ribosomal protein L14
IPI:IPI00745191.1	1	18.5	5.62	0.859	Similar to Dual specificity mitogen-activated protein kinase kinase 4
IPI:IPI00747893.1	1	26.1	9.66	0.858	Similar to DGCR14 protein
IPI:IPI00643237.1	3	15.4	8.59	0.858	Uncharacterized 15 kDa protein
IPI:IPI00556364.1	1	50.1	6.64	0.857	Interleukin enhancer binding factor 3 isoform c variant (Fragment)

IPI:IPI00479306.1	1	28.5	6.92	0.857	proteasome beta 5 subunit
IPI:IPI00217975.3	1	66.2	5.16	0.856	Lamin-B1
IPI:IPI00643932.1	19	70.0	5.66	0.856	heat shock 70kDa protein 1B
IPI:IPI00219525.9	1	53.0	7.23	0.855	6-phosphogluconate dehydrogenase, decarboxylating
IPI:IPI00419585.8	9	17.9	7.81	0.855	Peptidyl-prolyl cis-trans isomerase A
IPI:IPI00301699.1	1	17.0	9.23	0.854	Isoform 2 of Protein C6orf54
IPI:IPI00003362.2	18	72.4	5.16	0.854	78 kDa glucose-regulated protein
IPI:IPI00328736.5	3	293.2	7.66	0.854	ATP-binding cassette sub-family A member 12
IPI:IPI00642107.3	1	40.8	7.52	0.854	FNBP1 protein (Fragment)
IPI:IPI00026154.1	1	59.3	4.41	0.853	Glucosidase 2 beta subunit precursor
IPI:IPI00744939.1	2	26.1	9.79	0.853	Protein DBF4 homolog (Activator of S phase kinase)
IPI:IPI00013808.1	22	104.8	5.44	0.853	Alpha-actinin-4
IPI:IPI00375631.5	1	17.7	7.44	0.853	Interferon-induced 17 kDa protein precursor
IPI:IPI00015148.3	2	20.8	5.78	0.852	Ras-related protein Rap-1b precursor
IPI:IPI00428719.1	1	12.6	10.36	0.852	FP18315
IPI:IPI00011253.3	8	26.7	9.66	0.851	40S ribosomal protein S3
IPI:IPI00375380.3	1	39.8	6.30	0.850	proteasome 26S non-ATPase subunit 13 isoform 2
IPI:IPI00641363.1	3	279.5	5.05	0.848	Spectrin, alpha, erythrocytic 1 (red blood cell membrane)
IPI:IPI00016342.1	4	23.5	6.70	0.848	Ras-related protein Rab-7
IPI:IPI00399306.1	1	48.9	5.50	0.847	papillary renal cell carcinoma translocation-associated gene product isoform 2
IPI:IPI00339269.1	6	71.0	6.14	0.846	Heat shock 70 kDa protein 6
IPI:IPI00068653.1	1	32.0	8.85	0.846	CGI-93 protein
IPI:IPI00375513.3	1	24.4	5.33	0.845	Isoform Soluble of Catechol O-methyltransferase
IPI:IPI00220143.2	2	209.6	5.48	0.845	Maltase-glucoamylase, intestinal
IPI:IPI00657680.1	11	54.9	6.86	0.844	Uncharacterized 55 kDa protein
IPI:IPI00056318.3	1	24.0	4.21	0.844	Protein C6orf51
IPI:IPI00644359.1	3	104.7	6.51	0.843	Uncharacterized 105 kDa protein
IPI:IPI00002501.1	2	29.2	8.37	0.842	Cyclic AMP-dependent transcription factor ATF-1
IPI:IPI00027717.1	1	119.9	6.09	0.841	Component of gems 4

IPI:IPI00455843.1	1	63.9	9.00	0.841	PREDICTED: similar to transcription elongation factor B polypeptide 3 binding protein 1
IPI:IPI00051746.5	2	36.1	8.32	0.840	Olfactory receptor OR14-37
IPI:IPI00002186.3	1	202.0	6.49	0.840	Brefeldin A-inhibited guanine nucleotide-exchange protein 2
IPI:IPI00410272.2	1	77.8	5.03	0.839	Isoform 2 of Low-density lipoprotein receptor-related protein 8 precursor
IPI:IPI00641694.1	1	61.3	8.31	0.839	Ariadne-1 protein homolog variant (Fragment)
IPI:IPI00220989.1	1	54.3	9.52	0.838	Isoform 2 of Transcription factor SOX-30
IPI:IPI00010796.1	5	57.1	4.87	0.838	Protein disulfide-isomerase precursor
IPI:IPI00643454.1	1	53.4	8.95	0.835	Chromosome 9 open reading frame 93
IPI:IPI00383867.1	1	76.1	5.59	0.835	Polycystic kidney disease 2 related protein (Fragment)
IPI:IPI00746031.1	1	58.7	8.44	0.834	Similar to Receptor-type tyrosine-protein phosphatase V precursor
IPI:IPI00005826.1	5	527.1	6.27	0.834	E3 ubiquitin-protein ligase HERC2
IPI:IPI00031104.4	3	61.1	6.54	0.834	Mitochondrial protein with oncostatic activity (mitostatin, tumor suppressor protein)
IPI:IPI00642267.1	1	28.6	10.17	0.833	RPL3 protein
IPI:IPI00743966.1	1	5.6	8.19	0.833	Similar to Myosin light chain kinase, smooth muscle
IPI:IPI00651769.1	2	126.6	5.63	0.832	Myelin transcription factor 1-like protein
IPI:IPI00028954.1	1	218.3	6.39	0.832	80 kda MCM3-associated protein
IPI:IPI00478002.1	3	31.3	9.92	0.831	Uncharacterized 31 kDa protein
IPI:IPI00215918.2	4	20.4	7.14	0.831	ADP-ribosylation factor 4
IPI:IPI00745471.1	11	966.1	5.55	0.830	Spectrin-like protein of the nuclear envelope and Golgi
IPI:IPI00333264.7	2	93.4	7.06	0.829	Protein kinase A-anchoring protein 4, sperm motility
IPI:IPI00736299.1	2	118.2	7.96	0.824	Uncharacterized protein, similar to C28H8.3 isoform 8
IPI:IPI00440493.2	9	59.7	9.13	0.824	ATP synthase alpha chain, mitochondrial precursor
IPI:IPI00647546.1	1	16.1	11.09	0.823	16 kDa protein
IPI:IPI00005159.3	2	44.7	6.74	0.823	Actin-like protein 2
IPI:IPI00607647.1	1	35.5	9.14	0.823	Stomatin-like 1
IPI:IPI00217490.3	1	132.8	5.95	0.822	Factor for adipocyte differentiation 104 variant (Fragment)

IPI:IPI00645646.1	5	17.3	9.74	0.822	Solute carrier family 25
IPI:IPI00167909.4	1	95.1	7.12	0.822	EML3 protein
IPI:IPI00220364.1	1	49.0	7.09	0.822	Isoform Short of Mothers against decapentaplegic homolog 2
IPI:IPI00184409.6	1	63.4	7.11	0.821	Hypothetical protein DKFZp762G2015
IPI:IPI00419880.5	2	29.8	9.73	0.821	40S ribosomal protein S3a
IPI:IPI00640930.1	1	22.4	9.36	0.820	22 kDa protein
IPI:IPI00219018.6	15	35.9	8.46	0.819	Glyceraldehyde-3-phosphate dehydrogenase
IPI:IPI00013146.1	1	41.3	7.90	0.818	Mitochondrial 28S ribosomal protein S22
IPI:IPI00029144.1	2	130.2	5.21	0.818	Serine/threonine-protein phosphatase 2A 72/130 kDa regulatory subunit B
IPI:IPI00008529.1	2	11.7	4.54	0.817	60S acidic ribosomal protein P2
IPI:IPI00465431.6	2	26.0	8.56	0.817	Galectin-3
IPI:IPI00292892.3	1	146.1	6.61	0.816	KIAA1403 protein (Fragment)
IPI:IPI00411715.2	1	44.2	8.50	0.814	Isoform 2 of Nuclear factor NF-kappa-B p100 subunit
IPI:IPI00043669.1	1	32.1	9.74	0.813	CDNA FLJ30870 fis, clone FEBRA2004237
IPI:IPI00001451.1	1	87.0	4.87	0.813	Protocadherin beta 11 precursor
IPI:IPI00642345.1	1	36.0	9.01	0.813	LIM homeobox 9
IPI:IPI00335706.4	1	26.5	5.73	0.813	Isoform 2 of Dual specificity protein phosphatase 6
IPI:IPI00295098.3	1	29.7	9.04	0.813	Signal recognition particle receptor beta subunit
IPI:IPI00641837.1	1	27.3	9.10	0.812	27 kDa protein
IPI:IPI00465070.6	8	15.3	11.12	0.812	Histone H3.1
IPI:IPI00719814.1	1	5.7	9.70	0.812	ATP synthase, H+ transporting, mitochondrial F0 complex, subunit F2 isoform 2d
IPI:IPI00167009.3	1	42.1	9.20	0.810	CDNA FLJ38620 fis, clone HEART2007767
IPI:IPI00010405.3	2	104.2	7.17	0.810	Isoform Long of Tyrosine-protein kinase transmembrane receptor ROR1 precursor
IPI:IPI00306290.4	1	110.0	5.47	0.809	Exportin-T
IPI:IPI00397498.1	1	116.0	5.96	0.808	Isoform 2 of Carbamoyl-phosphate synthase [ammonia], mitochondrial precursor

IPI:IPI00642259.2	4	856.9	5.24	0.807	Bullous pemphigoid antigen 1, 230/240kDa, dystonin, Cytoskeletal linker protein
IPI:IPI00654777.2	2	39.1	5.45	0.806	Eukaryotic translation initiation factor 3 subunit 5
IPI:IPI00019385.1	1	19.0	6.15	0.805	Translocon-associated protein delta subunit precursor
IPI:IPI00164719.8	1	150.3	6.19	0.805	Connexin43-interacting protein of 150 kDa
IPI:IPI00746773.1	2	179.4	7.66	0.804	IQ motif containing GTPase activating protein 3
IPI:IPI00163085.2	3	118.0	7.64	0.804	Isoform 1 of Angiomotin
IPI:IPI00456969.1	3	532.1	6.40	0.804	Dynein heavy chain, cytosolic
IPI:IPI00303992.3	2	78.2	9.01	0.804	Vertebrae development associated vertinin
IPI:IPI00217223.1	1	49.6	7.49	0.803	Multifunctional protein ADE2
IPI:IPI00004671.1	3	375.8	5.00	0.803	Golgin subfamily B member 1
IPI:IPI00141938.3	5	12.1	10.46	0.801	H2A histone family, member V isoform 2
IPI:IPI00759647.1	1	36.6	9.03	0.801	BCL2-like 12 isoform 3
IPI:IPI00012268.3	5	100.1	5.20	0.800	26S proteasome non-ATPase regulatory subunit 2
IPI:IPI00003964.3	2	290.3	5.80	0.799	ubiquitin specific protease 9, X-linked isoform 4
IPI:IPI00028264.3	1	81.7	5.49	0.797	Exocyst complex component 8
IPI:IPI00329503.8	1	131.4	6.77	0.797	Transient receptor potential cation channel, subfamily M, member 5
IPI:IPI00160349.3	1	34.3	4.54	0.795	UPF0360 protein
IPI:IPI00328737.1	2	98.6	8.40	0.794	Zinc finger protein 598
IPI:IPI00397909.3	1	56.4	7.18	0.794	ankyrin repeat domain 36
IPI:IPI00747283.1	2	78.9	6.74	0.793	microtubule-associated protein tau isoform 1
IPI:IPI00006196.2	3	236.4	5.80	0.792	Isoform 2 of Nuclear mitotic apparatus protein 1
IPI:IPI00020984.1	6	67.5	4.60	0.792	Calnexin precursor
IPI:IPI00014375.1	2	109.2	5.50	0.790	Glutamyl aminopeptidase
IPI:IPI00333637.1	1	78.0	5.54	0.790	Isoform 2 of Golgin subfamily A member 5
IPI:IPI00025039.1	1	33.8	10.18	0.790	Fibrillarin
IPI:IPI00186439.7	1	49.9	9.35	0.790	Zinc finger protein 643
IPI:IPI00465189.1	2	34.1	9.76	0.789	Uncharacterized protein C11orf57
IPI:IPI00027252.6	4	33.3	9.83	0.789	Prohibitin-2

IPI:IPI00044004.1	1	58.8	6.20	0.788	WAP, follistatin/kazal, immunoglobulin, kunitz and netrin domain containing 1 precursor
IPI:IPI00745230.1	2	343.9	8.47	0.788	REV3-like, catalytic subunit of DNA polymerase zeta
IPI:IPI00657652.1	1	18.3	6.25	0.787	18 kDa protein
IPI:IPI00396435.2	1	92.8	7.25	0.786	DEAH (Asp-Glu-Ala-His) box polypeptide 15
IPI:IPI00007750.1	11	49.9	5.06	0.786	Tubulin alpha-1 chain
IPI:IPI00218918.4	14	38.6	7.02	0.784	Annexin A1
IPI:IPI00025427.1	1	18.4	10.24	0.784	Eosinophil cationic protein precursor
IPI:IPI00021485.2	1	80.7	6.15	0.784	Leucine-rich repeats neuronal protein 1 precursor
IPI:IPI00167598.1	2	51.2	5.20	0.783	Ankyrin repeat and SAM domain-containing protein 4B
IPI:IPI00748111.1	1	16.8	8.69	0.783	Isoform 2 of Mitoferrin-1
IPI:IPI00006599.1	1	13.9	5.22	0.782	Protein C1orf21
IPI:IPI00007052.6	1	16.9	8.79	0.782	Mitochondria fission 1 protein
IPI:IPI00025277.5	1	21.9	5.40	0.781	Programmed cell death protein 6
IPI:IPI00028055.4	1	25.0	7.44	0.780	Transmembrane emp24 domain-containing protein 10 precursor
IPI:IPI00152304.1	1	60.0	7.66	0.780	Hypothetical protein MGC27016
IPI:IPI00000897.1	2	218.8	7.37	0.779	Probable helicase with zinc-finger domain
IPI:IPI00215777.1	2	39.9	9.36	0.778	Isoform B of Phosphate carrier protein, mitochondrial precursor
IPI:IPI00442317.1	1	28.8	4.87	0.778	CDNA FLJ16159 fis, clone BRCAN2002854, weakly similar to Human Hsp27 ERE-TATA-binding protein (HET) mRNA
IPI:IPI00455562.2	1	70.5	9.06	0.776	PREDICTED: hypothetical protein LOC284371 isoform 2
IPI:IPI00386076.2	1	42.2	7.93	0.775	Isoform 2 of Tripartite motif protein 42
IPI:IPI00218450.1	1	98.4	8.03	0.774	Isoform 2 of Semaphorin-6C precursor
IPI:IPI00220834.7	3	82.5	5.81	0.773	ATP-dependent DNA helicase 2 subunit 2
IPI:IPI00619938.1	1	98.0	4.63	0.772	Rho GTPase activating protein 30 isoform 1
IPI:IPI00470434.1	2	55.7	7.05	0.770	Eyes absent homolog 2
IPI:IPI00554774.1	1	45.6	6.84	0.770	Isoform 2 of Receptor-interacting serine/threonine-protein kinase 2
IPI:IPI00013847.4	3	52.6	6.37	0.769	Ubiquinol-cytochrome-c reductase complex core protein I, mitochondrial precursor

IPI:IPI00410621.1	1	40.8	6.25	0.769	Isoform 2 of CCR4-NOT transcription complex subunit 2
IPI:IPI00021263.3	17	27.7	4.79	0.769	14-3-3 protein zeta/delta (Protein kinase C inhibitor protein 1)
IPI:IPI00513928.1	2	33.0	8.15	0.768	Isoform 2 of Acyl-coenzyme A thioesterase 2
IPI:IPI00032455.1	1	49.1	7.50	0.768	Zinc finger and BTB domain-containing protein 12
IPI:IPI00744826.1	3	69.6	9.45	0.767	Uncharacterized protein C10orf68
IPI:IPI00479630.2	1	59.9	6.77	0.765	fragile X mental retardation-related protein 1 isoform c
IPI:IPI00738582.1	1	22.2	6.40	0.764	PREDICTED: similar to junction-mediating and regulatory protein
IPI:IPI00397875.3	2	129.6	6.16	0.764	Simiar to tudor domain-containing protein 6
IPI:IPI00414676.5	14	83.1	5.03	0.762	Heat shock protein HSP 90-beta
IPI:IPI00442303.1	1	111.7	9.33	0.762	CDNA FLJ16186 fis, clone BRTHA2007060
IPI:IPI00641507.1	1	36.8	9.89	0.762	Transient receptor potential cation channel, subfamily M, member 3
IPI:IPI00719374.2	1	13.0	11.88	0.761	LOC441212 protein (Fragment)
IPI:IPI00418430.1	1	28.9	5.43	0.761	C6orf69 protein
IPI:IPI00217662.1	1	70.4	8.84	0.761	Hypothetical protein C11orf35
IPI:IPI00072918.2	1	322.0	6.90	0.760	alpha 3 type VI collagen isoform 4 precursor
IPI:IPI00386211.3	3	330.3	5.08	0.760	Biorientation of chromosomes in cell division protein 1-like 1
IPI:IPI00027107.5	5	49.8	7.61	0.760	Tu translation elongation factor, mitochondrial
IPI:IPI00165222.3	2	15.2	9.33	0.759	Uncharacterized 15 kDa protein
IPI:IPI00218743.1	1	61.6	8.88	0.755	Isoform 2 of Bestrophin-1
IPI:IPI00008137.1	1	118.8	8.29	0.755	Zinc finger protein 295
IPI:IPI00299155.5	1	29.5	7.72	0.755	Proteasome subunit alpha type 4
IPI:IPI00021405.3	26	74.1	7.02	0.755	Isoform A of Lamin-A/C
IPI:IPI00002459.4	2	75.2	5.67	0.752	Annexin VI isoform 2
IPI:IPI00429190.2	1	24.2	6.57	0.751	Ras-related protein Rab-11A
IPI:IPI00020153.2	2	416.1	7.55	0.749	Bassoon protein (Zinc finger protein 231)
IPI:IPI00009724.2	2	172.8	8.40	0.749	CAP-binding protein complex interacting protein 1 isoform a
IPI:IPI00335509.3	1	61.4	7.20	0.748	Dihydropyrimidinase-related protein 5
IPI:IPI00031047.1	3	51.9	6.52	0.747	IQ motif containing protein G

IPI:IPI00719669.1	2	17.0	4.36	0.747	Myosin regulatory light chain
IPI:IPI00383372.1	1	9.3	5.39	0.747	PRO1155
IPI:IPI00011528.1	1	48.3	6.58	0.746	Cleavage stimulation factor, 50 kDa subunit
IPI:IPI00738274.1	3	139.0	8.05	0.746	PREDICTED: similar to Complement C3 precursor
IPI:IPI00291922.2	2	26.4	4.79	0.745	Proteasome subunit alpha type 5
IPI:IPI00011727.2	1	42.9	9.17	0.743	Synaptotagmin-5
IPI:IPI00657936.1	1	116.6	6.40	0.742	Collagen, type XXVIII precursor
IPI:IPI00479934.1	2	15.1	8.92	0.741	Non-specific lipid-transfer protein (May play a role in regulating steroidogenesis)
IPI:IPI00216989.5	3	82.4	7.69	0.740	G protein-regulated inducer of neurite outgrowth 3
IPI:IPI00218592.3	1	22.3	7.94	0.740	Isoform ASF-3 of Splicing factor, arginine/serine-rich 1
IPI:IPI00414045.1	1	32.7	4.88	0.739	Cyclin D-binding Myb-like protein gamma
IPI:IPI00304809.6	2	151.0	6.71	0.738	Microtubule-associated tumor suppressor candidate 2
IPI:IPI00333016.5	1	57.2	7.53	0.738	DNAJC11 protein
IPI:IPI00168913.1	2	139.8	6.65	0.738	Isoform 2 of Limbin
IPI:IPI00005587.1	4	164.7	6.14	0.736	Myomesin-2
IPI:IPI00759663.1	5	17.0	7.24	0.735	Isoform Cytoplasmic/peroxisomal of Peroxiredoxin-5, mitochondrial precursor
IPI:IPI00004931.1	1	71.6	7.64	0.735	Nuclear RNA export factor 2
IPI:IPI00018206.3	3	47.4	9.01	0.734	Aspartate aminotransferase, mitochondrial precursor
IPI:IPI00008964.3	4	22.2	5.73	0.733	Ras-related protein Rab-1B
IPI:IPI00010214.1	1	11.7	5.24	0.732	Protein S100-A14
IPI:IPI00291006.1	8	35.5	8.68	0.732	Malate dehydrogenase, mitochondrial precursor
IPI:IPI00297084.7	2	50.8	6.55	0.731	Dolichyl-diphosphooligosaccharide-protein glycosyltransferase
IPI:IPI00303476.1	15	56.5	5.40	0.730	ATP synthase beta chain, mitochondrial precursor
IPI:IPI00163266.4	2	58.3	7.77	0.728	DEP domain-containing protein 7 (introcellular signal transduction)
IPI:IPI00016513.5	3	22.5	8.38	0.727	Ras-related protein Rab-10
IPI:IPI00014230.1	4	31.3	4.84	0.726	Complement component 1 Q subcomponent-binding protein, mitochondrial precursor

IPI:IPI00013890.1	19	27.8	4.74	0.725	14-3-3 protein sigma
IPI:IPI00009803.1	1	115.3	6.37	0.722	Integrin alpha-4 precursor
IPI:IPI00021828.1	3	11.1	7.56	0.720	Cystatin B
IPI:IPI00219038.8	7	15.2	11.27	0.718	Histone H3.3
IPI:IPI00646499.2	1	11.6	8.88	0.718	Similar to Splicing factor U2AF 35 kDa subunit
IPI:IPI00747988.1	1	29.5	4.37	0.715	30 kDa protein
IPI:IPI00641334.1	2	14.2	5.15	0.713	Cytochrome b5 type B precursor
IPI:IPI00657914.1	1	37.9	9.19	0.712	Isoform 2 of Armadillo repeat-containing X-linked protein 4
IPI:IPI00307551.3	1	172.6	8.10	0.712	Isoform A of Zinc finger protein 236
IPI:IPI00166612.9	2	448.9	4.78	0.711	Cardiomyopathy associated 5
IPI:IPI00743344.1	1	12.0	8.90	0.711	OTTHUMP00000021999
IPI:IPI00641665.1	1	12.4	5.17	0.709	12 kDa protein
IPI:IPI00059631.1	1	22.2	8.07	0.708	Hypothetical protein TMEM125
IPI:IPI00384444.4	22	51.5	5.16	0.706	Keratin, type I cytoskeletal 14
IPI:IPI00337766.5	3	91.7	7.39	0.705	zinc finger, CCHC domain containing 2
IPI:IPI00012465.1	1	41.6	7.71	0.705	Testis-specific serine/threonine-protein kinase 1
IPI:IPI00160716.2	1	89.3	8.21	0.704	DEP domain containing 1
IPI:IPI00450768.6	26	47.9	5.02	0.703	Keratin, type I cytoskeletal 17
IPI:IPI00060419.3	1	21.4	5.11	0.702	TRAF-interacting protein with a forkhead-associated domain
IPI:IPI00554711.1	8	81.4	6.38	0.702	Junction plakoglobin
IPI:IPI00297492.1	1	80.4	8.22	0.700	Oligosaccharyl transferase STT3 subunit homolog
IPI:IPI00017334.1	1	29.8	5.76	0.699	Prohibitin
IPI:IPI00514366.1	2	39.1	7.34	0.698	Mitochondrial carrier homolog 1
IPI:IPI00642900.2	3	241.1	7.05	0.697	Transcription repressor CCR4-NOT transcription complex subunit 1
IPI:IPI00011370.2	1	9.8	9.85	0.696	PRO1102
IPI:IPI00012966.1	1	72.9	7.02	0.692	Transcription factor 12
IPI:IPI00296676.2	4	98.9	8.92	0.692	Smoothelin isoform b
IPI:IPI00413614.3	1	74.5	7.50	0.692	Isoform 2 of Symplekin

IPI:IPI00217532.1	1	34.9	8.65	0.689	Isoform 2 of Sodium/potassium-transporting ATPase subunit beta-1
IPI:IPI00015911.1	1	54.1	7.68	0.684	Dihydrolipoyl dehydrogenase, mitochondrial precursor
IPI:IPI00166674.1	1	52.6	8.88	0.680	F-box only protein 39
IPI:IPI00386256.1	1	36.1	9.61	0.679	Pro protein (Fragment)
IPI:IPI00031514.1	1	38.5	7.20	0.677	Replication factor C subunit 5
IPI:IPI00747054.1	2	32.5	7.42	0.676	Tumor necrosis factor ligand superfamily member 10
IPI:IPI00016608.1	1	22.7	5.17	0.676	Transmembrane emp24 domain trafficking protein 2 precursor
IPI:IPI00396431.2	1	86.6	6.09	0.674	Isoform A of Ral guanine nucleotide dissociation stimulator-like 1
IPI:IPI00396243.5	2	151.7	6.35	0.673	Isoform 1 of WD-repeat protein 19 (cillia, cell projection)
IPI:IPI00736118.1	1	16.9	8.73	0.671	PREDICTED: similar to Dipeptidyl aminopeptidase-like protein 6
IPI:IPI00647556.1	1	25.8	7.08	0.669	Gelsolin
IPI:IPI00335277.1	1	26.4	7.23	0.669	Isoform 2 of Synaptophysin-like protein 1
IPI:IPI00220362.4	4	10.8	8.92	0.668	10 kDa heat shock protein, mitochondrial
IPI:IPI00465190.1	1	7.2	12.10	0.668	TCR gamma alternate reading frame protein isoform 1
IPI:IPI00739522.1	1	23.6	6.77	0.667	Isoform 2 of Keratinocytes-associated protein 3
IPI:IPI00332835.3	1	73.9	6.73	0.667	armadillo repeat containing 8 isoform 2
IPI:IPI00472102.3	14	61.2	5.87	0.666	Heat shock protein 60
IPI:IPI00384569.2	1	98.3	7.03	0.666	Hypothetical protein KIAA1685
IPI:IPI00022774.2	4	89.1	5.26	0.665	Transitional endoplasmic reticulum ATPase
IPI:IPI00477042.4	2	72.4	8.66	0.665	Uncharacterized protein CXorf57
IPI:IPI00742782.1	2	18.5	10.54	0.663	Uncharacterized 19 kDa protein
IPI:IPI00470829.2	2	80.0	6.74	0.662	Isoform 3 of Mitochondrial inner membrane protein
IPI:IPI00386437.1	1	41.3	9.38	0.660	SPATA22 protein
IPI:IPI00413456.2	1	98.5	6.65	0.658	Similar to elongation factor Tu GTP binding domain containing 1
IPI:IPI00438875.2	2	98.0	7.34	0.658	Isoform M6-kinase 2 of Transient receptor potential cation channel subfamily M member 6
IPI:IPI00015286.1	1	215.2	7.56	0.658	Dedicator of cytokinesis protein 1

IPI:IPI00607850.1	2	176.9	6.58	0.654	Isoform 3 of Citron Rho-interacting kinase
IPI:IPI00293575.7	1	86.1	8.07	0.653	Zinc finger protein 184
IPI:IPI00449139.1	1	58.1	9.67	0.653	LRFN4 protein (Fragment)
IPI:IPI00297169.1	1	60.2	6.27	0.652	Lymphocyte cytosolic protein 2
IPI:IPI00738097.1	3	48.5	5.25	0.652	tubulin, beta polypeptide 4, member Q
IPI:IPI00048232.1	1	13.4	8.44	0.651	Isoform A of Usher syndrome type-3 protein
IPI:IPI00384016.1	1	29.6	8.32	0.651	Full-length cDNA 5-PRIME end of clone CS0DJ009YL13 of T cells
IPI:IPI00034159.1	1	40.3	5.00	0.649	Vacuolar ATP synthase subunit d
IPI:IPI00640240.1	2	58.6	9.13	0.648	Serine palmitoyltransferase, long chain base subunit 2-like
IPI:IPI00218993.1	1	92.1	5.55	0.647	Isoform Beta of Heat-shock protein 105 kDa
IPI:IPI00299554.3	1	186.4	7.91	0.647	Kinesin-like protein KIF14
IPI:IPI00168769.6	3	222.4	7.81	0.647	Exophilin 5 (Rab GTPase binding)
IPI:IPI00401829.3	2	93.6	6.71	0.645	Coiled-coil domain-containing protein 175
IPI:IPI00455852.1	4	91.9	8.25	0.642	Isoform 1 of Rho guanine nucleotide exchange factor 15
IPI:IPI00718908.1	8	30.4	7.20	0.634	Isoform 6 of Voltage-dependent anion-selective channel protein 2
IPI:IPI00446003.1	1	15.5	12.21	0.632	CDNA FLJ43210 fis, clone FEBRA2020582
IPI:IPI00002966.1	3	94.2	5.26	0.632	Heat shock 70 kDa protein 4
IPI:IPI00216457.6	6	14.0	10.90	0.628	Histone H2A type 2-A
IPI:IPI00022750.5	1	21.4	12.22	0.627	Hypothetical protein (Fragment)
IPI:IPI00065310.2	3	75.3	5.68	0.626	Coiled-coil domain-containing protein 27
IPI:IPI00646947.1	3	95.1	5.77	0.626	highly similar to Sarcoplasmic/endoplasmic reticulum calcium ATPase 1
IPI:IPI00000816.1	4	29.2	4.74	0.625	14-3-3 protein epsilon
IPI:IPI00414858.3	2	94.0	5.57	0.617	component of golgi transport complex 3
IPI:IPI00014213.1	2	101.9	8.22	0.617	Probable leucyl-tRNA synthetase, mitochondrial precursor
IPI:IPI00009030.1	1	44.9	5.63	0.615	Isoform LAMP-2A of Lysosome-associated membrane glycoprotein 2 precursor
IPI:IPI00218131.2	2	10.4	6.25	0.613	Protein S100-A12
IPI:IPI00640715.2	1	103.2	7.15	0.607	KIAA1875 protein (Fragment)

IPI:IPI00259519.2	1	60.1	6.02	0.604	leucine rich repeat containing 22
IPI:IPI00744220.1	1	10.6	10.81	0.603	11 kDa protein
IPI:IPI00642269.1	1	14.3	8.38	0.603	CDNA FLJ38101 fis, clone D3OST1000270
IPI:IPI00657796.1	1	17.8	6.29	0.602	18 kDa protein
IPI:IPI00020124.1	1	54.0	8.29	0.601	Phosphatidylinositol 4-kinase type II
IPI:IPI00082310.1	1	44.0	7.28	0.600	Isoform 3 of KH domain-containing, RNA-binding, signal transduction-associated protein 1
IPI:IPI00249984.2	1	59.4	7.46	0.594	Isoform 2 of Death-inducer obliterator 1
IPI:IPI00219690.1	1	57.3	6.60	0.593	Isoform Beta-2 of Serine/threonine-protein phosphatase 2A 56 kDa regulatory subunit beta isoform
IPI:IPI00186338.1	2	9.7	6.09	0.592	Barrier-to-autointegration factor-like protein
IPI:IPI00009867.2	26	62.4	8.06	0.589	Keratin, type II cytoskeletal 5
IPI:IPI00025512.2	6	22.8	6.40	0.584	Heat-shock protein beta-1
IPI:IPI00736027.1	1	31.8	8.79	0.577	PREDICTED: similar to Succinyl-CoA ligase
IPI:IPI00294575.7	2	91.8	7.02	0.577	Cell division cycle protein 27 homolog
IPI:IPI00745977.1	1	15.2	10.14	0.576	Conserved hypothetical protein
IPI:IPI00334433.5	1	142.6	7.61	0.575	KIAA1454 protein (Fragment)
IPI:IPI00030874.1	1	19.2	5.58	0.575	Uncharacterized gastric protein YA42P (Fragment)
IPI:IPI00027273.1	2	178.2	5.57	0.571	Breast cancer 1, early onset isoform BRCA1-delta14-18
IPI:IPI00166863.2	1	89.5	6.80	0.569	hypothetical protein LOC84902
IPI:IPI00020991.2	2	61.1	9.01	0.568	CDKN2A interacting protein
IPI:IPI00154283.2	1	102.0	6.23	0.566	Hypothetical protein DKFZp762J0112
IPI:IPI00019007.1	1	103.8	7.18	0.566	Toll-like receptor 3 precursor
IPI:IPI00171611.5	8	15.4	11.27	0.563	H3 histone family, member M
IPI:IPI00646739.1	1	20.2	9.13	0.562	20 kDa protein
IPI:IPI00217185.1	4	551.0	5.64	0.561	Isoform 2 of Ryanodine receptor 3
IPI:IPI00174848.5	2	39.9	9.14	0.560	Similar to Ankyrin repeat domain protein 18A
IPI:IPI00166979.5	3	194.9	6.11	0.558	Leucine-rich repeat and WD repeat-containing protein KIAA1239
IPI:IPI00646867.1	4	49.6	5.25	0.555	Vimentin

IPI:IPI00022479.4	3	531.9	6.04	0.554	Guanine nucleotide exchange factor p532
IPI:IPI00022296.1	1	109.8	6.98	0.552	Mast/stem cell growth factor receptor precursor
IPI:IPI00007928.4	2	273.4	8.84	0.550	Pre-mRNA-processing-splicing factor 8
IPI:IPI00009634.1	4	49.9	9.11	0.546	Sulfide:quinone oxidoreductase, mitochondrial precursor
IPI:IPI00456697.1	1	35.9	6.92	0.545	SULT1C3 splice variant d
IPI:IPI00514369.1	2	38.7	11.06	0.544	Nuclear localized factor 2
IPI:IPI00759655.1	1	50.9	8.16	0.541	Isoform Cytoplasmic+peroxisomal of Malonyl-CoA decarboxylase, mitochondrial precursor
IPI:IPI00220327.2	8	65.8	8.12	0.538	Keratin, type II cytoskeletal 1
IPI:IPI00216308.4	9	30.6	8.54	0.534	Voltage-dependent anion-selective channel protein 1
IPI:IPI00743775.1	1	55.3	4.74	0.533	Isoform 2 of Coiled-coil domain-containing protein 47 precursor
IPI:IPI00015580.2	1	63.5	6.19	0.530	formin binding protein 1-like isoform 2
IPI:IPI00656138.1	1	60.6	5.76	0.519	Isoform 1 of Serine/threonine-protein kinase PAK 1
IPI:IPI00007797.2	4	15.0	7.01	0.517	Fatty acid-binding protein, epidermal
IPI:IPI00026015.1	1	57.4	6.64	0.515	Vesicular inhibitory amino acid transporter
IPI:IPI00426107.1	1	46.1	10.20	0.513	KI67 Antigen (Fragment)
IPI:IPI00746841.1	1	8.2	8.31	0.505	Similar to BCL2/adenovirus E1B 19-kDa protein-interacting protein 2
IPI:IPI00382471.1	1	13.7	8.41	0.502	Ig heavy chain V-I region WOL
IPI:IPI00657779.1	2	16.5	9.58	0.492	Peptidyl-prolyl cis-trans isomerase PPIF
IPI:IPI00061448.5	1	12.5	9.52	0.488	Hypothetical protein LOC90693
IPI:IPI00167383.2	3	87.4	5.07	0.487	PAS domain containing 1
IPI:IPI00185036.4	1	186.0	8.87	0.482	Myosin IIIA
IPI:IPI00021520.1	2	37.2	9.11	0.478	Glucocorticoid receptor AF-1 coactivator-1
IPI:IPI00376379.2	2	61.9	5.99	0.477	keratin 1B
IPI:IPI00552972.2	3	56.3	6.33	0.473	Dolichyl-diphosphooligosaccharide-protein glycosyltransferase subunit 2
IPI:IPI00168698.1	2	128.5	6.09	0.470	PDZ domain-containing protein 8
IPI:IPI00027412.4	2	37.2	5.82	0.470	Carcinoembryonic antigen-related cell adhesion molecule 6
IPI:IPI00007227.4	1	67.7	9.13	0.470	hypothetical protein LOC22864

IPI:IPI00002570.1	1	12.9	6.67	0.468	Eukaryotic translation initiation factor 4E-binding protein 2
IPI:IPI00658151.1	3	354.1	8.51	0.465	Isoform 1 of Striated muscle preferentially expressed protein kinase
IPI:IPI00450347.1	1	57.8	8.40	0.463	LOC197322 protein
IPI:IPI00043958.1	1	44.8	9.32	0.462	Isoform 3 of Fizzy-related protein homolog
IPI:IPI00001985.2	1	110.1	6.07	0.459	Isoform 1 of Vacuolar protein sorting 18
IPI:IPI00187002.8	2	137.8	9.16	0.456	Novel sickle tail protein homolog isoform 2
IPI:IPI00013933.1	15	331.6	6.81	0.454	Isoform DPI of Desmoplakin
IPI:IPI00741858.1	1	19.9	8.29	0.431	PREDICTED: similar to Protein FAM3C precursor
IPI:IPI00219886.1	1	122.1	5.34	0.428	Isoform Short of Ubiquitin carboxyl-terminal hydrolase 25
IPI:IPI00166301.3	1	84.1	6.67	0.410	CDNA FLJ33877 fis, clone CTONG2007072, weakly similar to N- CHIMAERIN
IPI:IPI00140420.4	1	101.9	7.17	0.408	Staphylococcal nuclease domain-containing protein 1
IPI:IPI00023208.1	1	54.0	7.49	0.404	Na/PO4 cotransporter homolog
IPI:IPI00022314.1	5	24.7	8.25	0.400	Superoxide dismutase [Mn], mitochondrial precursor
IPI:IPI00748646.1	1	9.0	11.41	0.388	Conserved hypothetical protein
IPI:IPI00619932.4	2	265.2	8.53	0.382	CREB-binding protein
IPI:IPI00288964.2	1	135.9	5.82	0.382	hypothetical protein LOC57719
IPI:IPI00006205.1	1	60.9	7.33	0.382	Acetyl-coenzyme A transporter 1
IPI:IPI00103259.2	1	22.5	10.15	0.371	HCV F-transactivated protein 1
IPI:IPI00219434.4	1	106.0	8.76	0.366	Disks large-associated protein 3
IPI:IPI00607843.1	1	21.0	9.09	0.356	OTTHUMP00000045524 (Fragment)
IPI:IPI00078069.1	1	62.5	10.59	0.355	transmembrane 7 superfamily member 2
IPI:IPI00009829.3	1	48.7	9.00	0.353	Mast cell carboxypeptidase A precursor
IPI:IPI00007611.1	3	23.3	9.96	0.353	ATP synthase O subunit, mitochondrial precursor
IPI:IPI00007047.1	2	10.8	7.03	0.351	Protein S100-A8
IPI:IPI00640590.1	1	67.9	5.52	0.349	Similar to ezrin-binding partner PACE-1 isoform 1
IPI:IPI00023711.1	4	231.5	6.96	0.348	Envoplakin
IPI:IPI00415040.1	1	33.8	6.76	0.344	Isoform 2 of ADP-ribose pyrophosphatase, mitochondrial precursor

IPI:IPI00006184.1	1	55.0	8.22	0.341	Monocarboxylate transporter 6
IPI:IPI00746049.1	3	131.1	6.20	0.326	Similar to Breast cancer antigen NY-BR-1.1
IPI:IPI00454970.4	1	57.7	7.94	0.326	PREDICTED: similar to notch1-induced protein
IPI:IPI00735374.1	1	75.7	9.35	0.323	Kruppel-like zinc finger protein isoform 1 (Fragment)
IPI:IPI00300725.6	26	59.9	8.00	0.311	Keratin, type II cytoskeletal 6A
IPI:IPI00441919.1	1	31.1	10.20	0.305	Hypothetical protein FLJ23970
IPI:IPI00024163.1	2	155.6	8.48	0.303	DNA-directed RNA polymerase III largest subunit
IPI:IPI00027462.1	6	13.2	6.13	0.297	Protein S100-A9
IPI:IPI00061168.2	1	41.0	4.97	0.295	Hypothetical protein DKFZp451K241
IPI:IPI00386402.1	1	14.2	10.07	0.291	CDNA FLJ13590 fis, clone PLACE1009398, moderately similar to ZINC FINGER PROTEIN 135
IPI:IPI00293867.6	1	12.6	7.30	0.287	D-dopachrome decarboxylase
IPI:IPI00022062.2	1	13.5	8.84	0.271	Small inducible cytokine A17 precursor
IPI:IPI00013888.1	1	29.7	5.80	0.266	Integral membrane protein 2A
IPI:IPI00304064.7	2	196.3	4.94	0.263	Isoform 1 of InaD-like protein
IPI:IPI00167663.1	1	43.8	5.36	0.262	CDNA FLJ38159 fis, clone DFNES2001404
IPI:IPI00018236.1	1	20.8	5.31	0.261	Ganglioside GM2 activator precursor
IPI:IPI00397683.2	1	59.8	10.24	0.255	CDNA FLJ40925 fis, clone UTERU2006486
IPI:IPI00329536.1	3	162.4	5.66	0.248	Early endosome antigen 1
IPI:IPI00300053.3	3	56.6	6.74	0.227	Keratin, type II cuticular Hb2
IPI:IPI00387050.3	2	55.5	9.57	0.216	Isoform 2 of BUD13 homolog (possibly splicing)
IPI:IPI00220081.1	1	147.4	7.05	0.158	Isoform 4 of Nuclear receptor coactivator 3
IPI:IPI00008359.1	7	65.8	8.12	0.154	Keratin, type II cytoskeletal 2 oral
IPI:IPI00396051.2	1	138.7	7.37	0.148	tensin like C1 domain containing phosphatase isoform 3
IPI:IPI00031547.1	3	107.4	5.00	0.146	Desmoglein-3 precursor
IPI:IPI00306170.5	1	72.7	10.05	0.125	Isoform 1 of GAS2-like protein 1
IPI:IPI00217963.2	24	51.1	5.05	0.121	Keratin, type I cytoskeletal 16
IPI:IPI00300594.3	1	65.3	8.70	0.119	CDNA FLJ35435 fis, clone SMINT2002620
IPI:IPI00217842.4	2	67.6	9.14	0.116	Hypothetical protein LOC114825 (function unknown)
IPI:IPI00015557.3	2	17.7	6.01	0.097	Coiled-coil domain-containing protein 48

IPI:IPI00218310.3	1	26.2	6.52	0.096	Exosome complex exonuclease RRP41
IPI:IPI00376143.1	2	60.6	6.25	0.029	minichromosome maintenance protein 7 isoform 2
